# Enzymatic Synthesis of Rhamnose Containing Chemicals by Reverse Hydrolysis

**DOI:** 10.1371/journal.pone.0140531

**Published:** 2015-10-27

**Authors:** Lili Lu, Qian Liu, Lan Jin, Zhenhao Yin, Li Xu, Min Xiao

**Affiliations:** 1 State Key Lab of Microbial Technology and National Glycoengineering Research Center, Shandong Provincial Key Laboratory of Carbohydrate Chemistry and Glycobiology, Shandong University, Jinan 250100, PR China; 2 Academy of State Administration of Grain, Beijing 100037, PR China; University of Insubria, ITALY

## Abstract

Rhamnose containing chemicals (RCCs) are widely occurred in plants and bacteria and are known to possess important bioactivities. However, few of them were available using the enzymatic synthesis method because of the scarcity of the α-L-rhamnosidases with wide acceptor specificity. In this work, an α-L-rhamnosidase from *Alternaria* sp. L1 was expressed in *Pichia pastroris* strain GS115. The recombinant enzyme was purified and used to synthesize novel RCCs through reverse hydrolysis in the presence of rhamnose as donor and mannitol, fructose or esculin as acceptors. The effects of initial substrate concentrations, reaction time, and temperature on RCC yields were investigated in detail when using mannitol as the acceptor. The mannitol derivative achieved a maximal yield of 36.1% by incubation of the enzyme with 0.4 M L-rhamnose and 0.2 M mannitol in pH 6.5 buffers at 55°C for 48 h. In identical conditions except for the initial acceptor concentrations, the maximal yields of fructose and esculin derivatives reached 11.9% and 17.9% respectively. The structures of the three derivatives were identified to be α-L-rhamnopyranosyl-(1→6')-D-mannitol, α-L-rhamnopyranosyl-(1→1')-β-D-fructopyranose, and 6,7-dihydroxycoumarin α-L-rhamnopyranosyl-(1→6')-β-D-glucopyranoside by ESI-MS and NMR spectroscopy. The high glycosylation efficiency as well as the broad acceptor specificity of this enzyme makes it a powerful tool for the synthesis of novel rhamnosyl glycosides.

## Introduction

Oligosaccharides are widely distributed in nature and play important roles in many processes, such as membrane-structure modulation, cell-cell recognition, communication, adhesion and viral infections [1–5]. Currently, the synthesis of oligosaccharides has attracted worldwide attention either for theoretical studies on their functions *in vivo* or for practical applications in the food and medical industries [6–8]. Traditional chemical synthesis requires complicated steps to control the stereo- and regio-specificities of the reaction [9–12]. In contrast, the enzymatic synthesis technique presents several practical advantages. Two classes of enzymes, namely glycosyltransferases (EC 2.4) and glycosidases (EC 3.2.1), can catalyze the formation of a specific glycosidic bond, in one step and under environment-friendly conditions [13]. For the large-scale production of oligosaccharides, glycosidases are more advantageous since they are readily available and tolerate simple, inexpensive donor substrates in comparison to the glycosyltransferases, which need high-cost donor substrates [14].

Glycosidases catalyze the synthesis of oligosaccharides via transglycosylation (kinetically controlled process) or reverse hydrolysis (thermodynamically controlled process) [15–16]. In transglycosylation reactions, the enzymes catalyze the transfer of sugar residues from oligosaccharides or glycoside donors into acceptor substrates thereby forming glycoside products. These reactions are widely used for oligosaccharides synthesis catalyzed by glycosidases. However, by-reactions of donor hydrolysis and self-transglycosylation usually occur simultaneously within the transglycosylation process, resulting in unwanted by-products. By-products can affect the transglycosylation efficiency and render necessary more purification steps in order to obtain the desired products. In contrast, the reverse hydrolysis reactions employ monosaccharides as donors, therefore by-reactions of donor hydrolysis cannot happen. In addition, the self-condensation of donors during reverse hydrolysis rarely occurs in the presence of acceptors. The relevant reaction mixtures, including donor, acceptor and glycoside products, are more easily purified. Moreover, the monosaccharide donors used for reverse hydrolysis are significantly cheaper than their artificial glycoside donors used in transglycosylation reactions. In recent years, several glycosidases such as β-glucosidase, β-galactosidase, α-mannosidase and endo-α-*N*-acetylgalactosaminidase have been used to synthesize glycosides, mainly alkyl glycosides, through reverse hydrolysis [17–20].

α-L-Rhamnosidases (EC 3.2.1.40) are an important class of glycosidases that can hydrolyze the terminal, non-reducing L-rhamnose residues from many natural products and catalyze the synthesis of RCCs by reverse hydrolysis [21, 22]. Rhamnose-rich oligo- and polysaccharides obtained from *Klebsiella pneumoniae* and *Klebsiella planticola* cultures are notable RCCs considered to have a protective effect on normal human dermal fibroblasts against AGE-induced cytotoxicity (AGE, advanced glycation end-products). This suggested potential therapetic applications against hyperglycemia induced cytotoxic effects as in type II-diabetes [23]. Also notable are two rhamnose containing glycosides of long-chain fatty alcohols, 1-*O*-[α-L-Rha(1→2)β-D-Glc(1→3)α-L-Rha(1→6)β-D-Glc]hexadecanol and 1-*O*-[α-L-Ara(1→3)α-L-Rha(1→2)β-D-Glc(1→3)α-L-Rha(1→6)β-D-Glc]hexadecanol, obtained from the stem bark of *Dimocarpus fumatus*, that exhibited *in vitro* cytotoxic activity against oral epithelial carcinoma KB cells [24]. Another interesting finding is that the rhamnose moiety of steroidal alkaloids could affect the binding specificity to steroid receptors and triggering death of human hepatoma cell (Hep3B) by apoptosis [25].

Despite the importance of RCCs, very few rhamnosyl chemicals have been synthesized with the enzymatic method compared to other glycosides, e.g. the galactoside and glucoside. This might be attributed to the scarcity of the α-L-rhamnosidases with wide acceptor specificity. To date, only the α-L-rhamnosidases from *Aspergillus niger*, *Aspergillus terreus*, *Penicillium decumbens* and *Penicillium ulaiense* have been used to glycosylate glucose and glucoside via reverse hydrolysis [22]. Although the enzyme from *A*. *terreus* was recently extended to glycosylate aliphatic and aromatic alcohols, the product yields were rather modest, never exceeding 5% (w/w) [26].

In our previous work, a novel strain of *Alternaria* sp. L1 with naringin hydrolysis activity was isolated from a rotten orange, and an α-L-rhamnosidase gene (*rha*L1) was obtained and expressed on the cell surface of *Saccharomyces cerevisiae* EBY-100 for naringin hydrolysis [27]. In this work, the *rha*L1 gene was cloned into the vector pPIC9K and expressed in *Pichia pastoris* GS115 of which the enzyme could be extracellularly excreted and easy for purification as pure biocatalyst. The purified recombinant RhaL1 was found to catalyze glycosylation efficiently utilizing L-rhamnose as glycosyl donor via reverse hydrolysis, resulting in a series of novel rhamnose containing chemicals.

## Materials and Methods

### Strains and Plasmid

*Pichia pastoris* GS115 and the plasmid pPIC9k were obtained from Multi-Copy *Pichia* Expression Kit (Invitrogen, USA). All the media mentioned below were prepared according to the protocols in the Multi-Copy *Pichia* Expression Kit (Invitrogen, USA).

### Expression and purification of α-L-rhamnosidase from *Pichia pastroris*

The α-L-rhamnosidase (RhaL1) gene from *Alternaria* sp. L1 had been cloned in our previous work with GenBank accession no. JN704640. In this study, the amplification of the gene was carried out using pPIC9k-F (5’-GCCTACGTATCAACGCCCTACTCTC-3’) and pPIC9k-R (5’-ACTGCGGCCGCCTCCGAACATCTAAAC-3’) as primers. The *SnaB* I and *Not* I restriction sites were underlined. The PCR product was digested and then ligated into plasmid pPIC9K. The recombinant plasmid pPIC9K/*rhaL1* was linearized by *Sal* I and then electroporated into *P*. *pastoris* GS115. The transformants were selected by the growth on the MD agar plates after incubation for 2–3 days at 30°C and screened again by PCR using yeast cells as templates.

The correct transformants were cultivated at 28°C in 10 mL BMGY medium for enzyme expression. Cells were harvested at the density of 2.0–5.0 at 600 nm and then cultivated in 50 mL BMMY medium. Methanol was supplemented to a final concentration of 0.5% (v/v) every 24 h to keep the induction. After 5 to 6 days, the culture was centrifugated at 15,777 x g by Thermo scientific Sorvall ST 16R centrifuge for 5 min and the supernatant was precipitated by addition of 80% ammonium sulfate. The resulting precipitate was subsequently resuspended, desalted and concentrated by ultrafiltration (50 kDa cutoff membrane, Millipore) to reach electrophoretical purity.

### Enzyme Assay

α-L-Rhamnosidase activity was measured by addition of 5 μL enzyme to 300 μL of 2 mM *p*-nitrophenyl-α-L-rhamnoside (*p*NPR). The reaction was performed at 37°C for 10 min and then stopped by addition of 700 μL of 5 mM Na_2_CO_3_. The amount of *p*-nitrophenol released was measured at 400 nm. One unit of enzyme activity (U) was defined as the amount of enzyme required to liberate 1 μM of *p*-nitrophenol per minute under the assay conditions.

### Biochemical studies

The optimal pH was assayed by incubating the enzyme with 2 mM *p*NPR in 30 mM buffers containing citric acid, KH_2_PO_4_, boric acid and barbitone and using NaOH to adjust the pH from 2.5 to 12.0. The effect of pH on enzyme stability was determined by incubation in the same range at 4°C for 24 h. The optimal temperature was determined by exposing the samples to temperatures ranging from 25 to 70°C for 10 min. Thermal stability was studied by assessing enzyme activity after incubation at the above temperatures for 2 h.

### Synthesis of RCCs by α-L-rhamnosidase

RCCs synthesis reactions were initially performed using L-rhamnose as glycosyl donor and three representative compounds (D-mannitol, D-fructose, and esculin) as acceptors. The influence of variations in the process parameters on RCCs synthesis were investigated in detail using 0.5 U of enzyme in 50 μL reaction mixture (pH 6.5). The products derived from D-mannitol, D-fructose, and esculin were designated as RCC-I, RCC-II, and RCC-III, respectively.

The effects of variations in donor concentrations (0.1–2 M) were investigated in the presence of 0.8 M D-manntiol at 55°C for 18 h. The effects of temperature and reaction time were investigated by incubating the enzyme with 0.4 M L-rhamnose and 0.8 M D-manntiol at 40 to 65°C and analyzing the mixture sequentially for 72 h. The influence of initial acceptor concentrations on RCC-I synthesis were determined by incubating the enzyme with 0.4 M L-rhamnose and mannitol at the concentrations ranging from 0.04 M to 1.6 M at 55°C for 48 h.

RCC-II and RCC-III synthesis followed the same optimal conditions found for RCC-I production except for the acceptor concentrations. The effects of acceptor concentrations were assayed by incubating the enzyme with 0.05–1.4 M fructose or 0.01–0.1 M esculin in the presence of 0.4 M L-rhamnose at 55°C for 48 h. All the reactions were stopped by heating at 100°C for 10 min. Samples were analyzed with a high-performance liquid chromatographic system (HPLC).

### Isolation and purification of RCCs

The reaction products of RCCs were separated by a Bio-Gel P2 (Bio-Rad, US) column (1.6 × 90 cm) with distilled water as the eluent. The fractions were eluted at a flow rate of 0.4 mL/min and collected at 1.5 mL per tube. The resulting samples were spotted on the thin layer chromatography (TLC) plate and detected directly by spraying a solution composed of 0.5% (w/v) 3,5-dihydroxytoluene in 20% (v/v) sulfuric acid and heating at 120°C for 5 min. The samples appearing colored sugars were loaded on TLC plate again, and separated using n-butanol:ethanol:water (5:3:2, v/v/v) as the mobile phase. The fractions with identical sugar compositions were combined and concentrated to dry powder.

### HPLC and TLC analysis

HPLC was performed using an Agilent ZORBAX carbohydrate column. Samples were filtered through a 0.22 μm polypropylene filter. The mobile phase containing acetonitrile/water (7:3, v/v) was degassed in an ultrasonic bath before use. Analysis was carried out with a Agilent 1200 refractive index detector using a column oven temperature of 30°C and a flow rate of 1 mL/min. TLC was performed by loading samples on Silica gel 60 F254 plates (Merck, Germany). The developing solvent was a mixture of *n*-butanol:ethanol:water (5:3:2, v/v/v). Sugars on the TLC plate were detected by spraying a solution composed of 0.5% (w/v) 3,5-dihydroxytoluene in 20% (v/v) sulfuric acid and heating at 120°C for 5 min.

### MS and NMR analysis

Mass spectra were recorded on a Shimadzu LCMS-IT-TOF instrument (Kyoto, Japan) equipped with an ESI source in positive/negative ion mode at a resolution of 10,000 full width at half-maximum. ^1^H and ^13^C NMR spectra were recorded at 25°C with a Bruker DRX Avance 600 MHz spectrometer (Switzerland) at 600 MHz for ^1^H and 150 MHz for ^13^C. Chemical shifts in parts per million (ppm) were reported relative to the internal standard 2,2-dimethyl-2-silapentane-5-sulfonate. Chemical shifts and coupling constants were obtained from first- and second-order spectra analysis. Standard homo- and hetero-nuclear correlated 2D techniques were used to support the assignments, including correlation spectroscopy (COSY), heteronuclear single quantum coherence (HSQC), and heteronuclear multiple band correlation (HMBC) experiments.

### Spectrometric identification of RCCs

α-L-Rhamnopyranosyl-(1→6')-D-mannitol (RCC-I). ESI-MS: [M + Na]^+^ 351.1. ^1^H NMR (600 MHz, D_2_O): *δ* 4.73 (1H, *J*_C1,H1_ = 174.7 Hz, H-1), 3.92 (1H, H-2), 3.73 (1H, H-3), 3.36 (1H, H-4), 3.66 (1H, H-5), 1.22 (3H, H-6), 3.78 (1H, H-1'_a_), 3.59 (1H, H-1'_b_), 3.67 (1H, H-2'), 3.70 (1H, H-3'), 3.76 (1H, H-4'), 3.78 (1H, H-5'), 3.89 (1H, H-6'_a_), 3.52 (1H, H-6'_b_). ^13^C NMR (150 MHz, D_2_O): *δ* 100.3 (C-1), 69.9 (C-2), 70.1 (C-3), 72.0 (C-4), 68.5 (C-5), 16.6 (C-6), 63.2 (C-1'), 70.7 (C-2'), 69.1 (C-3'), 68.9 (C-4'), 69.4 (C-5'), 69.1 (C-6').

α-L-Rhamnopyranosyl-(1→1')-β-D-fructopyranose (RCC-II). ESI-MS: [M + Na]^+^ 349.1. ^1^H NMR (600 MHz, D_2_O): *δ* 4.68 (1H, *J*_C1,H1_ = 177.3 Hz, H-1), 3.86 (1H, H-2), 3.68 (1H, H-3), 3.29 (1H, H-4), 3.60 (1H, H-5), 1.14 (3H, H-6), 3.46 (2H, H-1'), 3.85 (1H, H-3'), 3.75 (1H, H-4'), 3.61 (1H, H-5'), 3.88 (1H, H-6'_a_), 3.54 (1H, H-6'_b_). ^13^C NMR (150 MHz, D_2_O): *δ* 100.1 (C-1), 69.6 (C-2), 69.8 (C-3), 71.8 (C-4), 68.5 (C-5), 16.3 (C-6), 69.1 (C-1'), 97.6 (C-2'), 68.8 (C-3'), 69.1 (C-4'), 67.8 (C-5'), 63.4 (C-6').

6,7-Dihydroxycoumarin α-L-rhamnopyranosyl-(1→6')-6-β-D-glucopyranoside (RCC-III). ESI-MS: [M + Na]^+^ 509.4. ^1^H NMR (600 MHz, D_2_O): *δ* 4.69 (1H, *J*_C1,H1_ = 169.8 Hz, H-1), 3.80 (1H, H-2), 3.66 (1H, H-3), 3.30 (1H, H-4), 3.58 (1H, H-5), 1.06 (3H, H-6), 4.91 (1H, H-1'), 3.55 (1H, H-2'), 3.44 (1H, H-3'), 3.64 (1H, H-4'), 3.55 (1H, H-5'), 3.94 (1H, H-6'_a_), 3.64 (1H, H-6'_b_), 7.61 (1H, H-2''), 6.12 (1H, H-3''), 7.03 (1H, H-5''), 6.59 (1H, H-8''). ^13^C NMR (150 MHz, D_2_O): *δ* 100.3 (C-1), 69.9 (C-2), 70.1 (C-3), 72.0 (C-4), 68.5 (C-5), 16.4 (C-6), 101.0 (C-1'), 75.2 (C-2'), 69.3 (C-3'), 74.9 (C-4'), 72.7 (C-5'), 66.3 (C-6'), 164.4 (C-1''), 145.4 (C-2''), 111.6 (C-3''), 111.6 (C-4''), 114.2 (C-5''), 142.3 (C-6''), 150.7 (C-7''), 103.6 (C-8''), 150.3 (C-9'').

## Results and Discussion

### Expression and purification of α-L-rhamnosidase in *Pichia pastroris*

**Fig 1:**
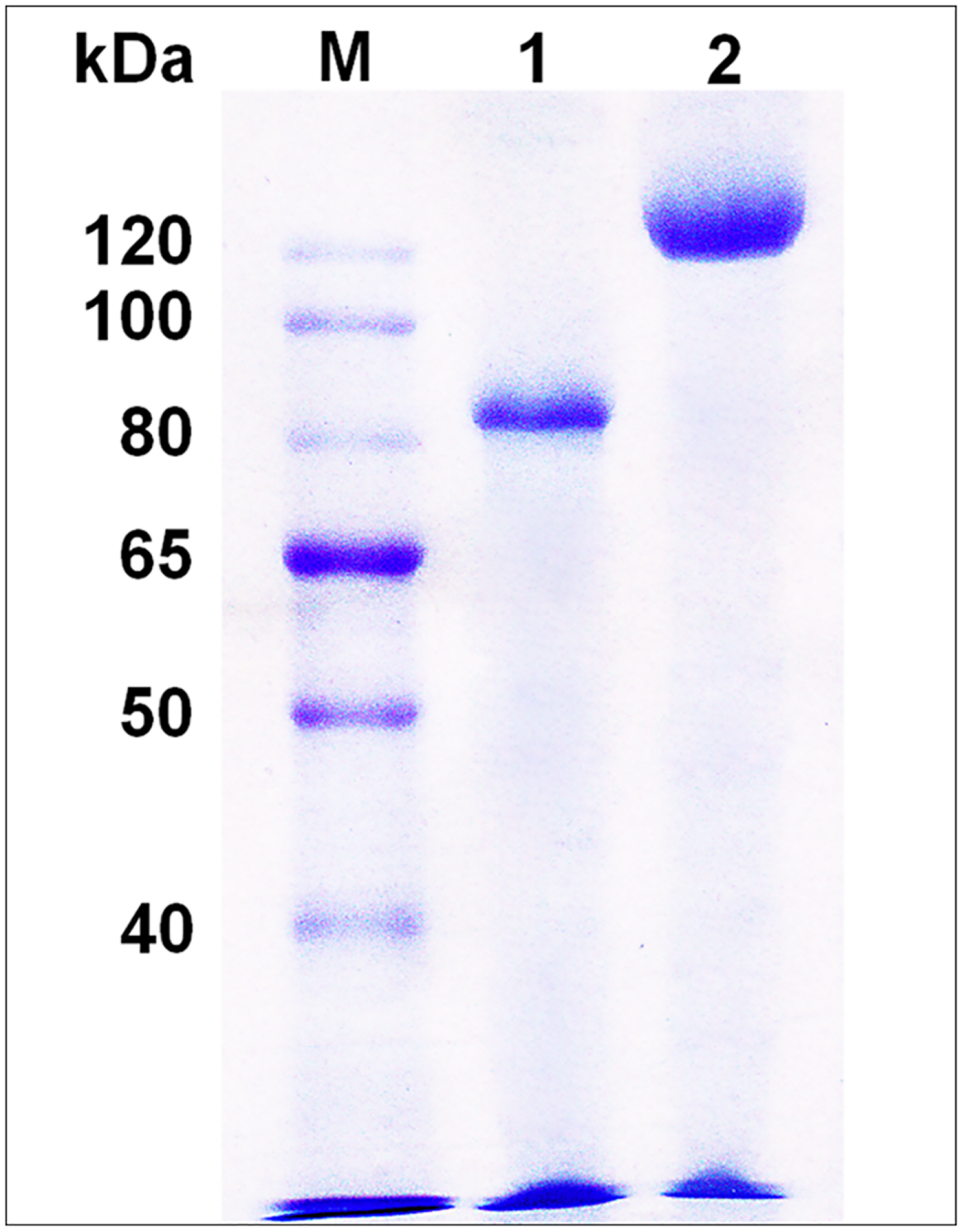
SDS-PAGE of the purified recombinant RhaL1 and PNGase F-treated RhaL1. Lane 1, PNGase F-treated RhaL1; Lane 2, purified recombinant RhaL1; M, molecular mass markers.

The α-L-rhamnosidase gene (*rha*L1) was cloned into the vector pPIC9K and expressed in *P*. *pastoris* GS115. With the addition of methanol in the culture, the extracellular α-L-rhamnosidase activity was increased during the prolonged incubation time. It reached a maximum of 2,270 U/L at 144 h when the total α-L-rhamnosidase protein achieved 237 mg/L. The recombinant α-L-rhamnosidase was purified to homogeneity from the culture. The final protein displayed a single band on SDS-PAGE and the molecular mass was calculated to be about 120 kDa (Fig 1). Since the sequence of RhaL1 was predicted to contain 19 potential *N*-glycosylation sites in the previous work [27], the enzyme was subsequently treated with PNGase F to remove *N*-glycans. The PNGase F can cleave the amide bond between the proximal *N*-acetylyglucosamine and the asparagine residue on glycopeptides or glycoproteins [28]. The molecular mass of the enzyme devoid of *N*-glycans reduced to about 85 kDa, confirming the existence of *N*-glycosylation sites in the protein.

### Biochemical studies

**Fig 2:**
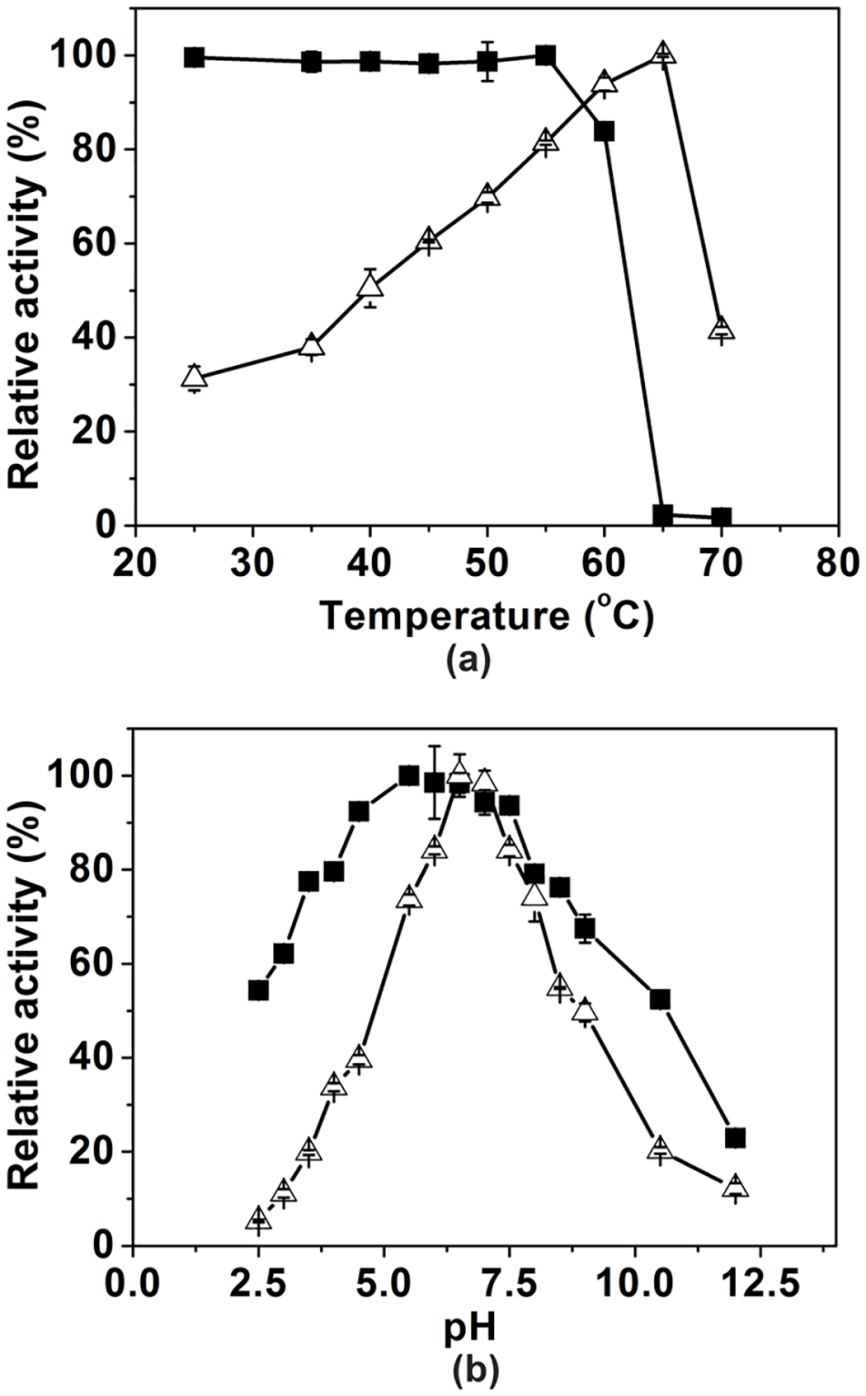
Effects of temperature (a) and pH (b) on activity (Δ) and stability (■) of recombinant RhaL1. Data points represent the means ± S.D. of three replicates.

The recombinant RhaL1 activity was determined at different temperature and pH. The enzyme was highly active at 60 to 65°C, and could keep above 80% activity after 2 h incubation below 60°C (Fig 2a); the optimal pH for enzyme activity was 6.5 to 7.0, and the enzyme was stable between pH 4.0 and 8.0 (Fig 2b). The good stability of the recombinant RhaL1 at high temperatures and within a broad pH range means that it is a particularly good candidate for use as a biocatalyst.

### Synthesis of rhamnosyl mannitol (RCC-I) by α-L-rhamnosidase

Rhamnosyl mannitol was synthesized by the recombinant RhaL1 using L-rhamnose as the glycosyl donor and mannitol as the acceptor. The influences of variations in substrate concentrations, reaction time, and temperature on RCC-I yield were evaluated. Samples were analyzed by HPLC and the yield was defined as the percentage of rhamnoside in the product mixture.

**Fig 3:**
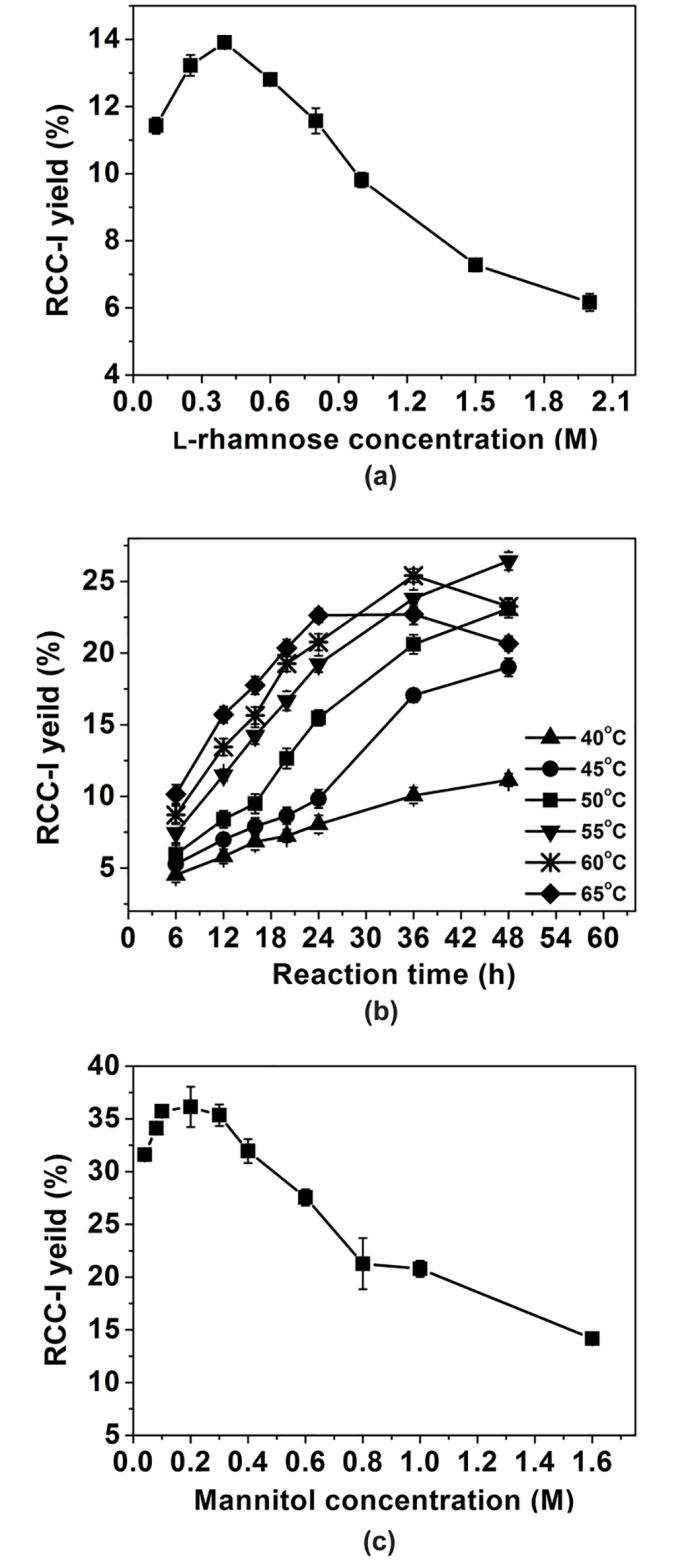
Effects of reaction conditions on RCC-I synthesis. (a) initial L-rhamnose concentrations; (b) temperature and reaction time; (c) initial mannitol concentrations. Data points represent the means ± S.D. of three replicates.

The yields of the mannitol derivative (RCC-I) were improved as L-rhamnose concentrations were increased from 0.1 to 0.4 M before declining at higher L-rhamnose levels. The maximum yield reached about 14.0% (w/w) at 0.4 M L-rhamnose (Fig 3a). The influences of temperature and reaction time on RCC-I synthesis were determined (Fig 3b). To a certain extent, the reactions could proceed more quickly at higher temperatures. The RCC-I yields kept increasing with increasing temperature from 40 to 55°C. The reaction incubated at 55°C for 48 h displayed a maximal yield of 26.4%. The yield then decreased with increasing incubation time (data not shown). Mannitol concentrations ranging from 0.04 to 1.60 M were subsequently tested (Fig 3c). The yield of the RCC-I was apparently improved from 31.6% to 35.7% with the increase of the mannitol concentrations from 0.04 to 0.1 M. This was followed by a more modest improvement with the yield reaching a maximum of 36.1% at 0.2 M.

In summary, the optimum conditions for RCC-I synthesis were a final L-rhamnose concentration of 0.4 M, a final mannitol concentration of 0.2 M, and 48 h incubation at 55°C. The product was separated and analyzed by MS and NMR spectroscopy.

The positive-ion ESI-mass spectrum of RCC-I showed a peak of [M+Na]^+^ ion at *m/z* 351.1 (S1 Fig), which was in accordance with the molecular mass of rhamnosyl mannitol (328). The complete structural characterization of the RCC-I was retrieved from the results of 2D NMR analysis involving ^1^H-^1^H COSY, HSQC and HMBC experiments, which were used to assign the chemical shifts of the sugar residues present in RCC-I (S2–S8 Figs). The proton signal of the peak at *δ* 4.73 ppm in the ^1^H NMR spectrum and the carbon signal at *δ* 100.3 ppm in the ^13^C spectrum were considered to be the H-1 and C-1 of the rhamnose residue. That was further confirmed by their correlation signals in the HSQC spectrum. The coupling constant of C-1 and H-1 of the rhamnose unit (*J*_C1,H1_) was 174.7 Hz, indicating an α-form of the anomeric carbon [27]. The protons at *δ* 3.78 and 3.59 ppm coupled with the same carbon at *δ* 63.2 ppm in the HSQC spectrum were identified as H-1's and C-1' signals of the mannitol residue. Cross peaks were found between C-1 (*δ* 100.3 ppm) of the rhamnose residue and H-6's (*δ* 3.89 ppm, *δ* 3.52 ppm) of the mannitol residue in the HMBC spectrum. These results demonstrated that the chemical structure of RCC-I was α-L-rhamnopyranosyl-(1→6')-D-mannitol.

### Synthesis of rhamnosyl fructose (RCC-II) by α-L-rhamnosidase

**Fig 4:**
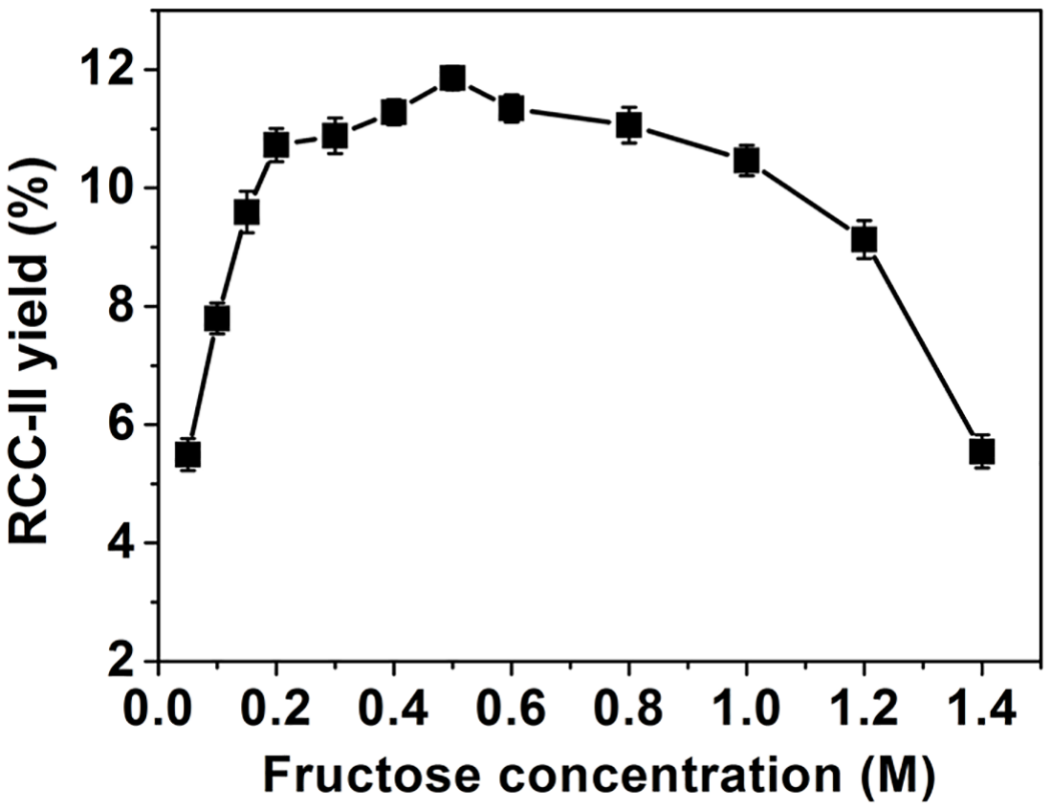
Effects of initial fructose orconcentrations on RCC-II synthesis. Data points represent the means ± S.D. of three replicates.

The synthesis of RCC-II was performed according to the optimal conditions of RCC-I production except for the final acceptor concentration. The fructose concentrations ranging from 0.05 to1.4 M were tested in the presence of 0.4 M L-rhamnose. The maximum yield of RCC-II was about 11.9% at 0.5 M fructose (Fig 4). The RCC-II was purified and analyzed by MS and NMR spectroscopy (S9–S16 Figs).

The positive-ion ESI-mass spectrum of RCC-II showed a peak of [M+Na]^+^ ion at *m/z* 349.1, which was in accordance with the molecular mass of rhamnosyl fructose (326). An obvious peak of the anomeric proton were found at *δ* 4.68 ppm in the ^1^H NMR spectrum while an anomeric carbon signal was located at *δ* 100.1 ppm in the ^13^C spectrum. They showed correlation signals in the HSQC spectrum and were identified as the H-1 and C-1 of the rhamnose residue. The C-2' of another sugar residue located at *δ* 97.6 ppm in ^13^C NMR spectrum. It showed correlation signals with H-6' proton in HMBC, suggesting that the fructose existed as a fructopyranose conformation. Cross peaks were found between C-1 (*δ* 100.1 ppm) of the rhamnose residue and H-1' (*δ* 3.46 ppm) of the fructose residue in the HMBC spectrum. Together with the coupling constant of C-1 and H-1 of rhamnose (*J*_C1,H1_ = 177.3 Hz), the chemical structure of RCC-II was determined to be α-L-rhamnopyranosyl-(1→1')-β-D-fructopyranose.

### Synthesis of rhamnosyl esculin (RCC-III) by α-L-rhamnosidase

**Fig 5:**
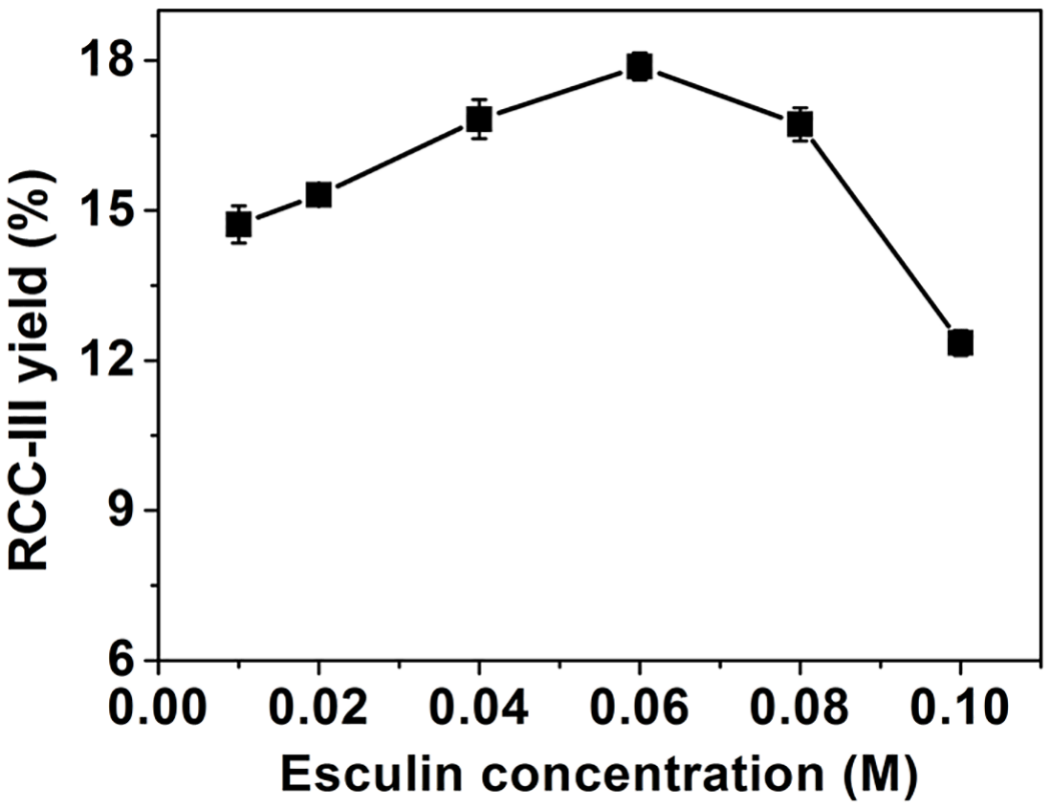
Effects of initial esculin concentrations on RCC-III synthesis. Data points represent the means ± S.D. of three replicates.

The RCC-III synthesis reaction was also carried out following the optimal conditions of RCC-I production except for the initial acceptor concentration. The maximum yield was achieved with 17.9% at 0.06 M esculin (Fig 5). The RCC-III was purified and analyzed by MS and NMR spectroscopy (S17–S24 Figs).

**Fig 6:**
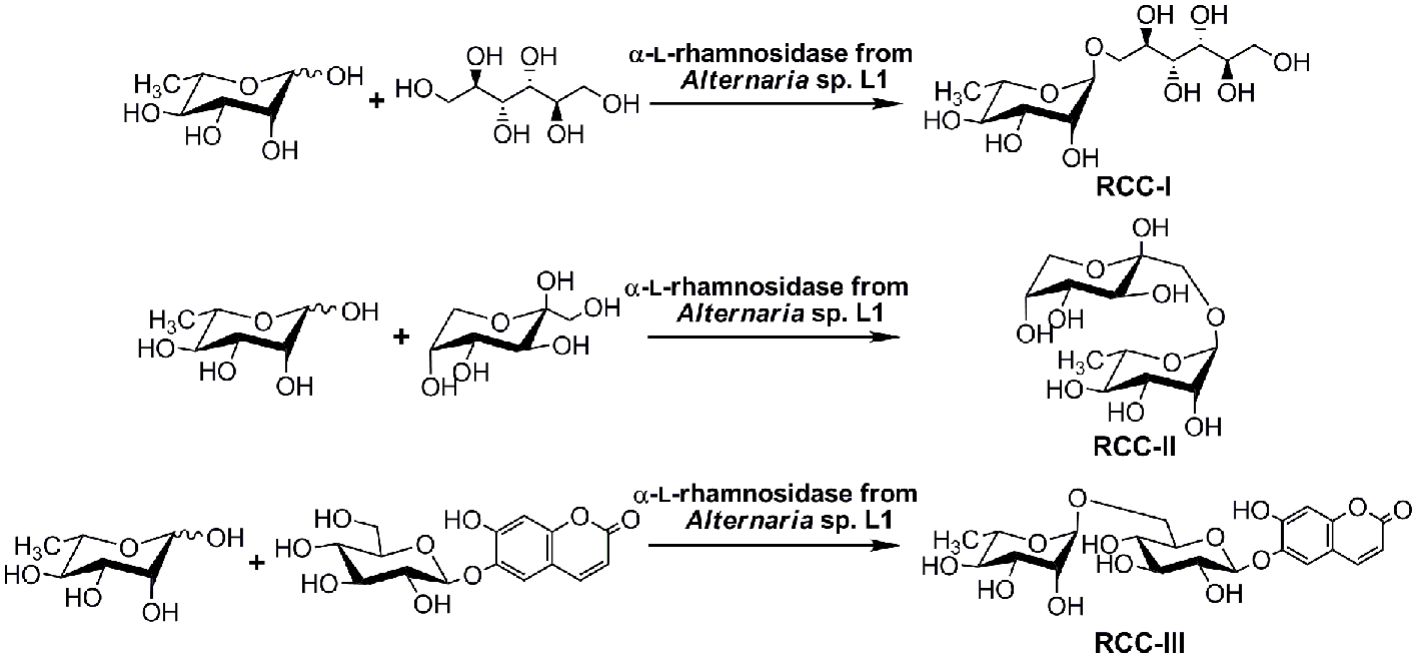
RCCs syntheses by the recombinant RhaL1 via reverse hydrolysis. The recombinant enzyme synthesized novel RCCs in the presence of L-rhamnose as donor and mannitol, fructose or esculin as acceptors.

The positive-ion ESI-mass spectrum of RCC-III showed a peak of [M+Na]^+^ ion at *m/z* 509.4, which was in accordance with the molecular mass of rhamnosyl esculin (486). The α-form of the rhamnose residue was determined based on its H-1 peak at 4.69 ppm as well as the coupling constant of C-1 and H-1 (*J*_C1,H1_ = 169.8 Hz) in the HSQC spectrum. The H-1 (*δ* 4.69 ppm) of the rhamnose residue and the C-6' (*δ* 66.3 ppm) of the glucose residue showed clear correlation signals in the HMBC spectrum, indicating an α-(1→6)-linkage between the two sugar residues. The proton signal of the peak at *δ* 4.91 ppm in the ^1^H NMR spectrum and the carbon signal at *δ* 101.0 ppm in the ^13^C spectrum were considered to be the H-1' and C-1' of the glucose residue. The cross peak between the H-1' of glucose (*δ* 4.91 ppm) and the C-6'' of esculetin (*δ* 142.3 ppm) confirmed the (1→6)-linkage in the esculin acceptor. Based on these results, the RCC-III structure was determined to be 6,7-dihydroxycoumarin α-L-rhamnopyranosyl-(1→6')-6-β-D-glucopyranoside. The chemical structures of all the above RCCs synthesized by the recombinant RhaL1 are shown in Fig 6.

## Conclusions

This paper provides a convenient method for synthesis of novel rhamnosyl glycoside derivatives by glycosidase-mediated reverse hydrolysis. The α-L-rhamnosidase from *Alternaria* sp. L1 was found to efficiently catalyze the formation of glycosidic linkage between monosaccharide donor and various acceptors. The relatively low process cost and the wide acceptor tolerance suggested that this method could potentially be implemented at an industrial scale to prepare more RCCs with desirable inherent bioactivity or as intermediates for further valuable modifications.

## Supporting Information

S1 Fig MS spectrum of RCC-I (*M*_*r*_ *328*). (TIF)

S2 Fig ^1^H NMR spectrum of RCC-I. (TIF)

S3 Fig ^13^C NMR spectrum of RCC-I. (TIF)

S4 Fig DEPT90 spectrum of RCC-I. (TIF)

S5 Fig DEPT135 spectrum of RCC-I. (TIF)

S6 Fig COSY spectrum of RCC-I. (TIF)

S7 Fig HSQC spectrum of RCC-I. (TIF)

S8 Fig HMBC spectrum of RCC-I. (TIF)

S9 Fig MS spectrum of RCC-II (*M*_*r*_ *326*). (TIF)

S10 Fig ^1^H NMR spectrum of RCC-II. (TIF)

S11 Fig ^13^C NMR spectrum of RCC-II. (TIF)

S12 Fig DEPT90 spectrum of RCC-II. (TIF)

S13 Fig DEPT135 spectrum of RCC-II. (TIF)

S14 Fig COSY spectrum of RCC-II. (TIF)

S15 Fig HSQC spectrum of RCC-II. (TIF)

S16 Fig HMBC spectrum of RCC-II. (TIF)

S17 Fig MS spectrum of RCC-III (*M*_*r*_ *486*). (TIF)

S18 Fig ^1^H NMR spectrum of RCC-III. (TIF)

S19 Fig ^13^C NMR spectrum of RCC-III. (TIF)

S20 Fig DEPT90 spectrum of RCC-III. (TIF)

S21 Fig DEPT135 spectrum of RCC-III. (TIF)

S22 Fig COSY spectrum of RCC-III. (TIF)

S23 Fig HSQC spectrum of RCC-III. (TIF)

S24 Fig HMBC spectrum of RCC-III. (TIF)
