# Supplementary figures and images for: Enzymatic Synthesis of Rhamnose Containing Chemicals by Reverse Hydrolysis
Source: PLoS One. 2015 Oct 27;10(10):e0140531. doi: 10.1371/journal.pone.0140531 (PMC4624630; doi:10.1371/journal.pone.0140531)

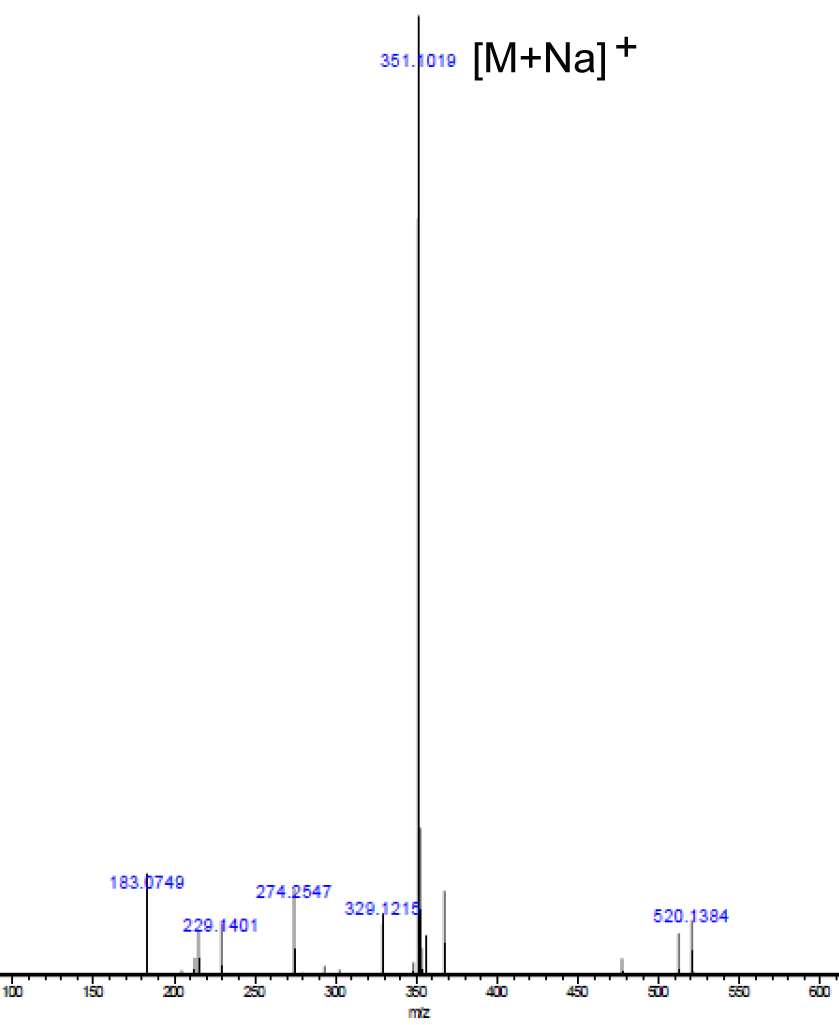

Supplement: S1 Fig — (TIF) [file pone.0140531.s001.tif]

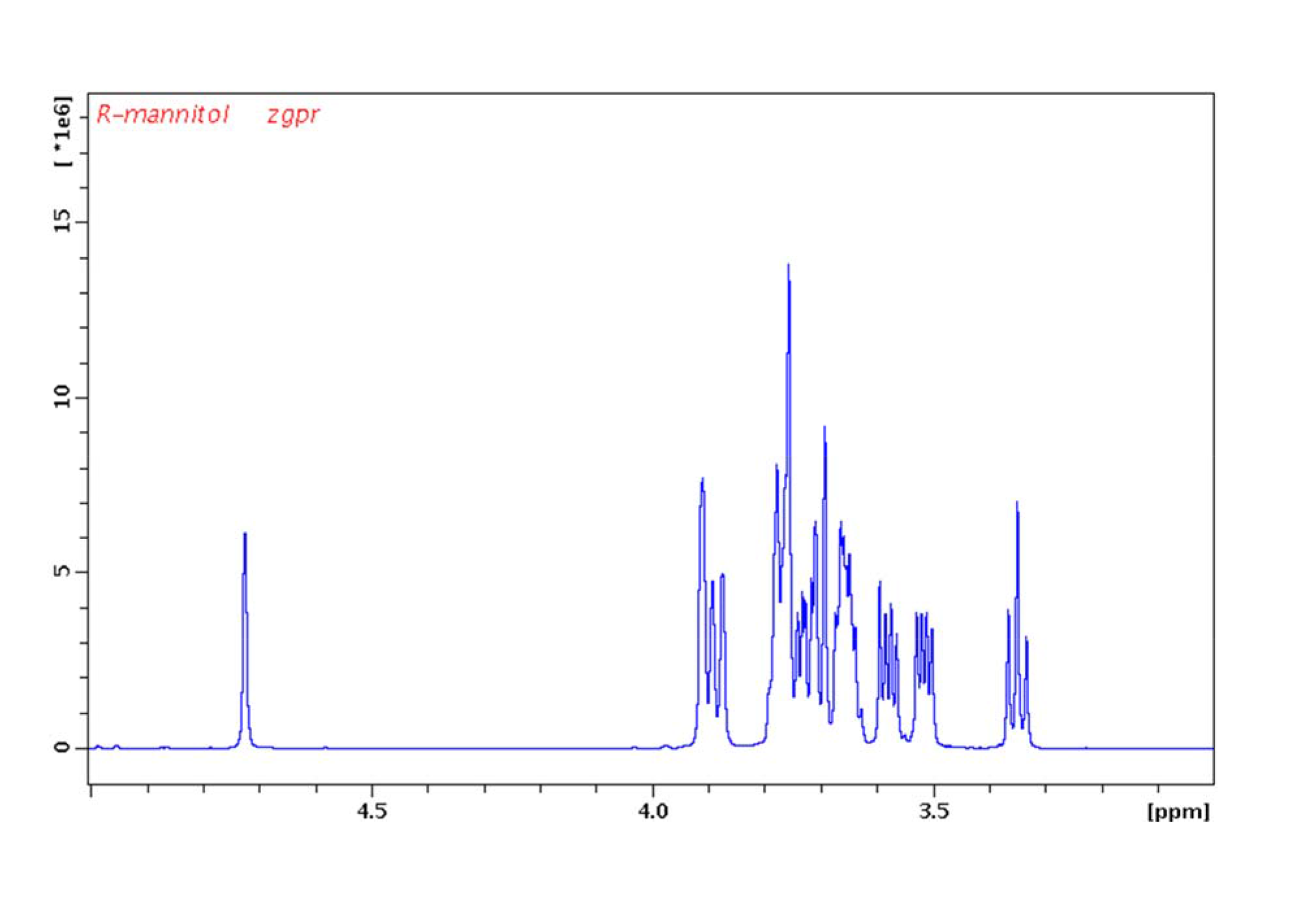

Supplement: S2 Fig — (TIF) [file pone.0140531.s002.tif]

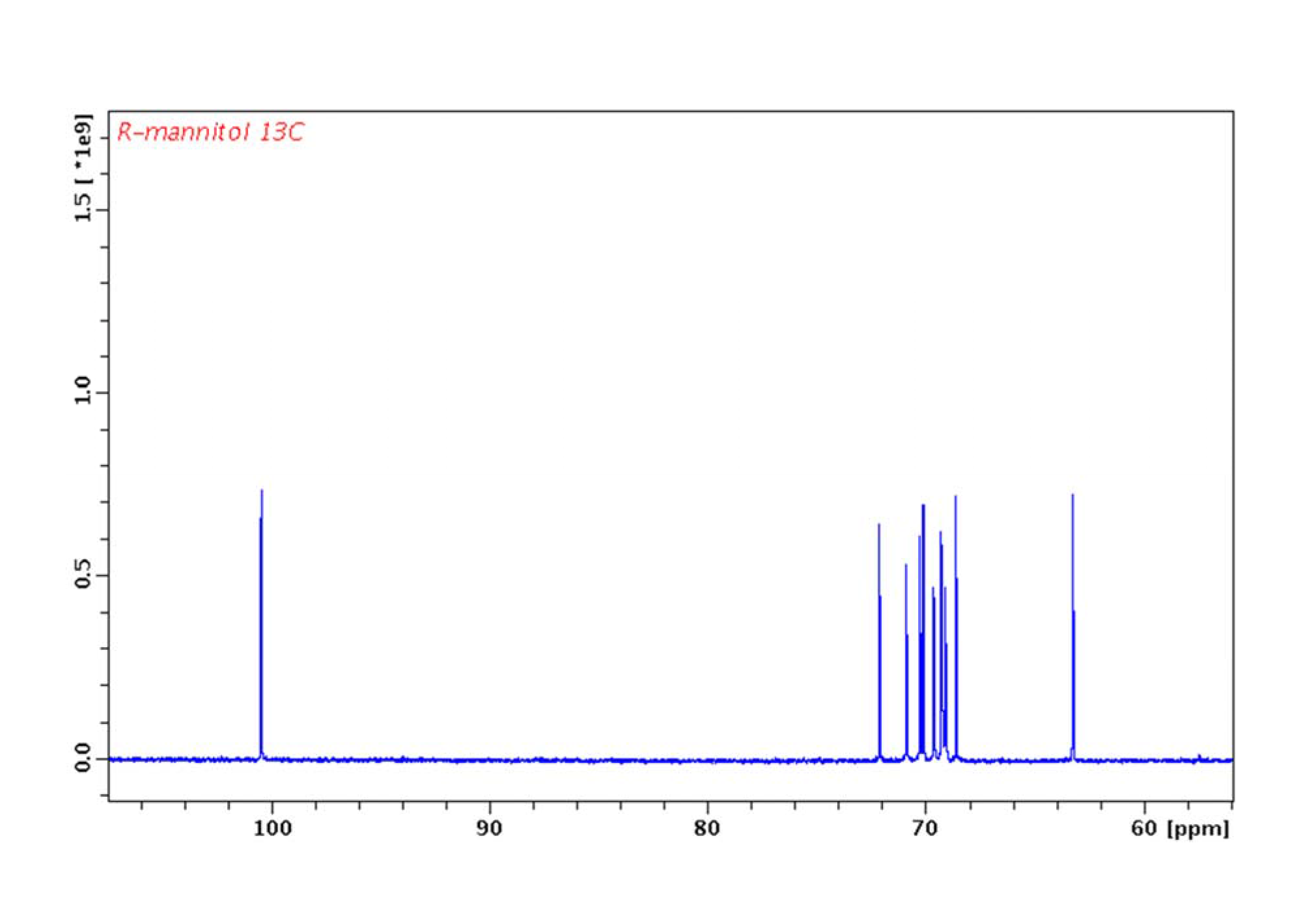

Supplement: S3 Fig — (TIF) [file pone.0140531.s003.tif]

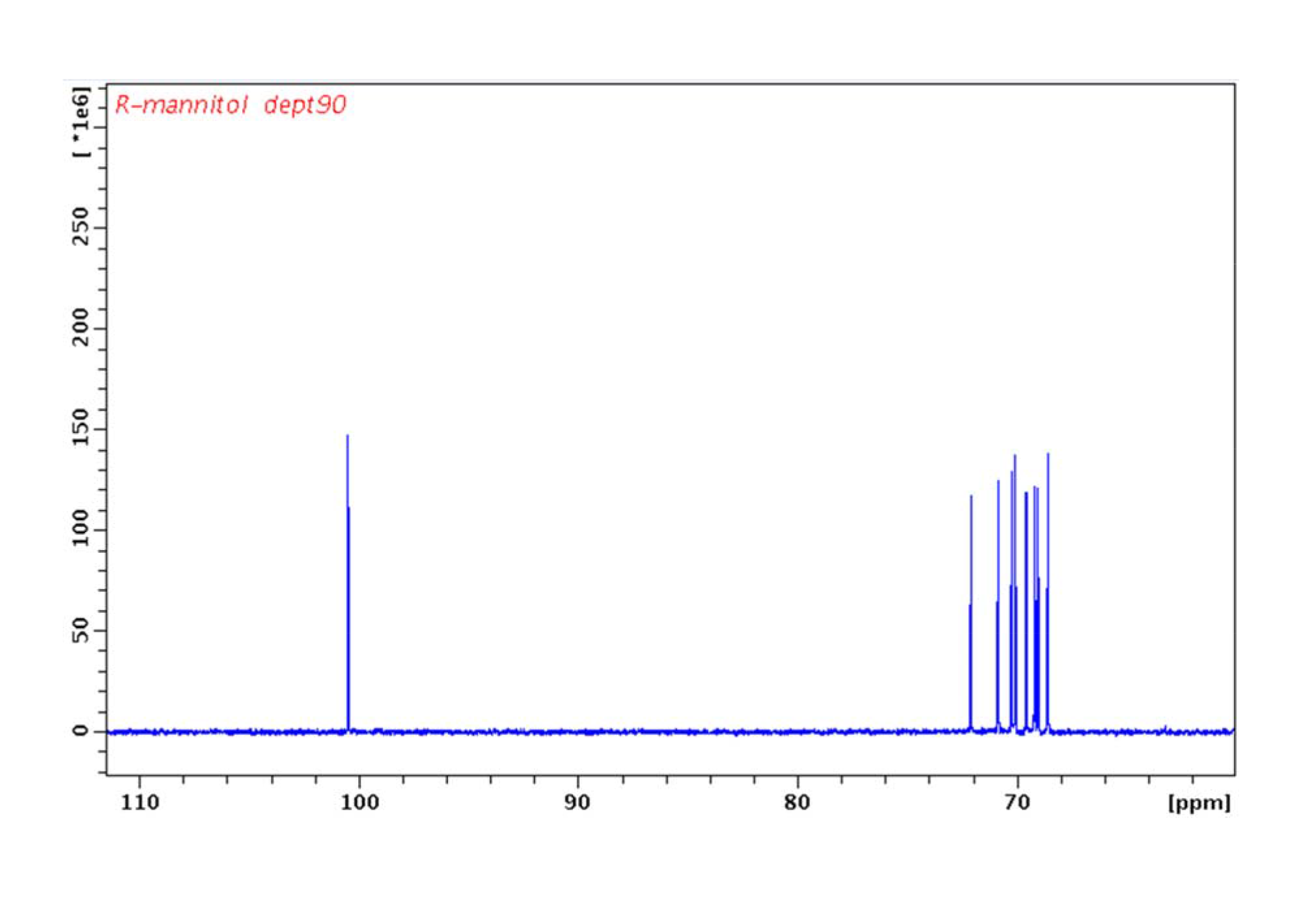

Supplement: S4 Fig — (TIF) [file pone.0140531.s004.tif]

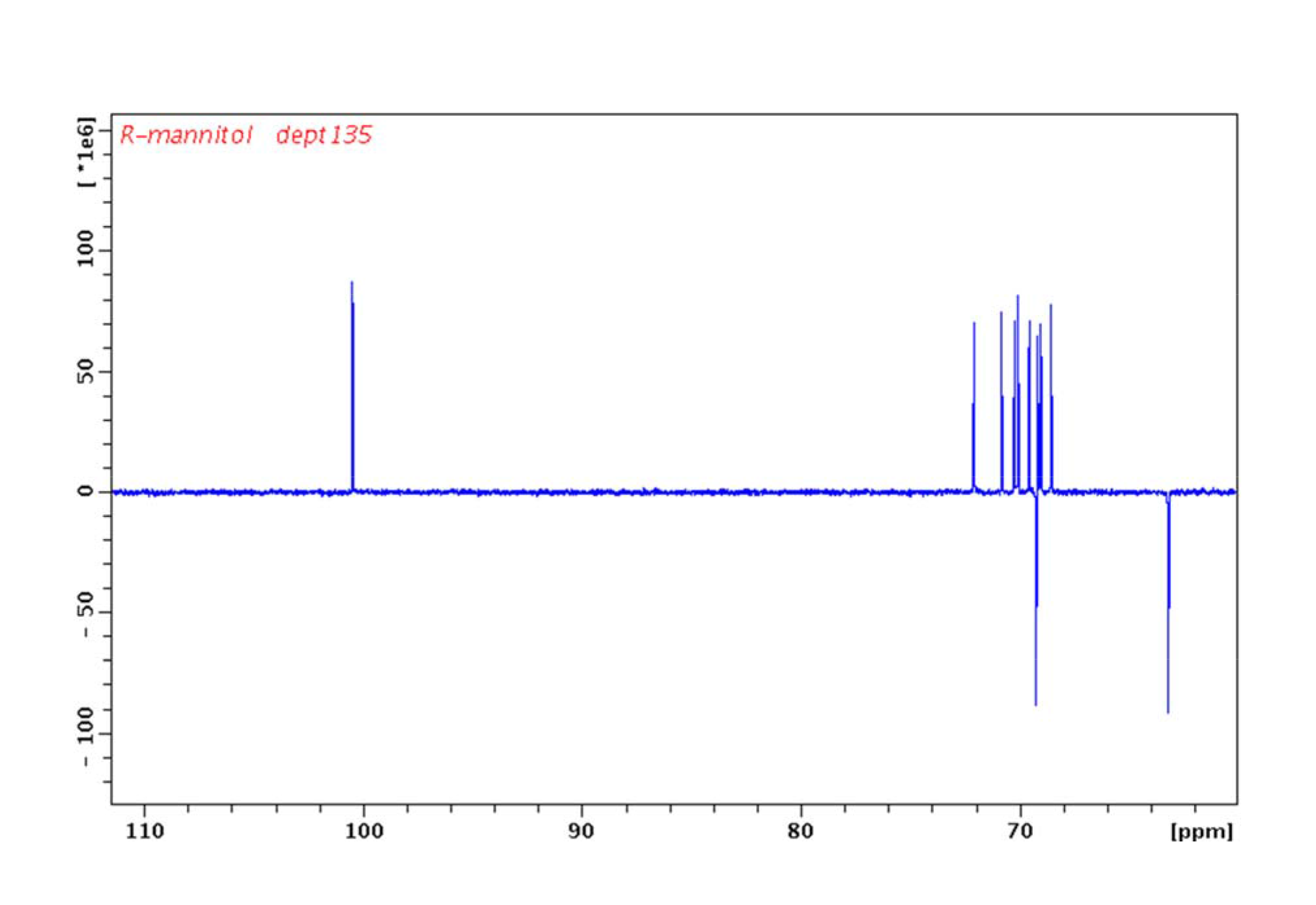

Supplement: S5 Fig — (TIF) [file pone.0140531.s005.tif]

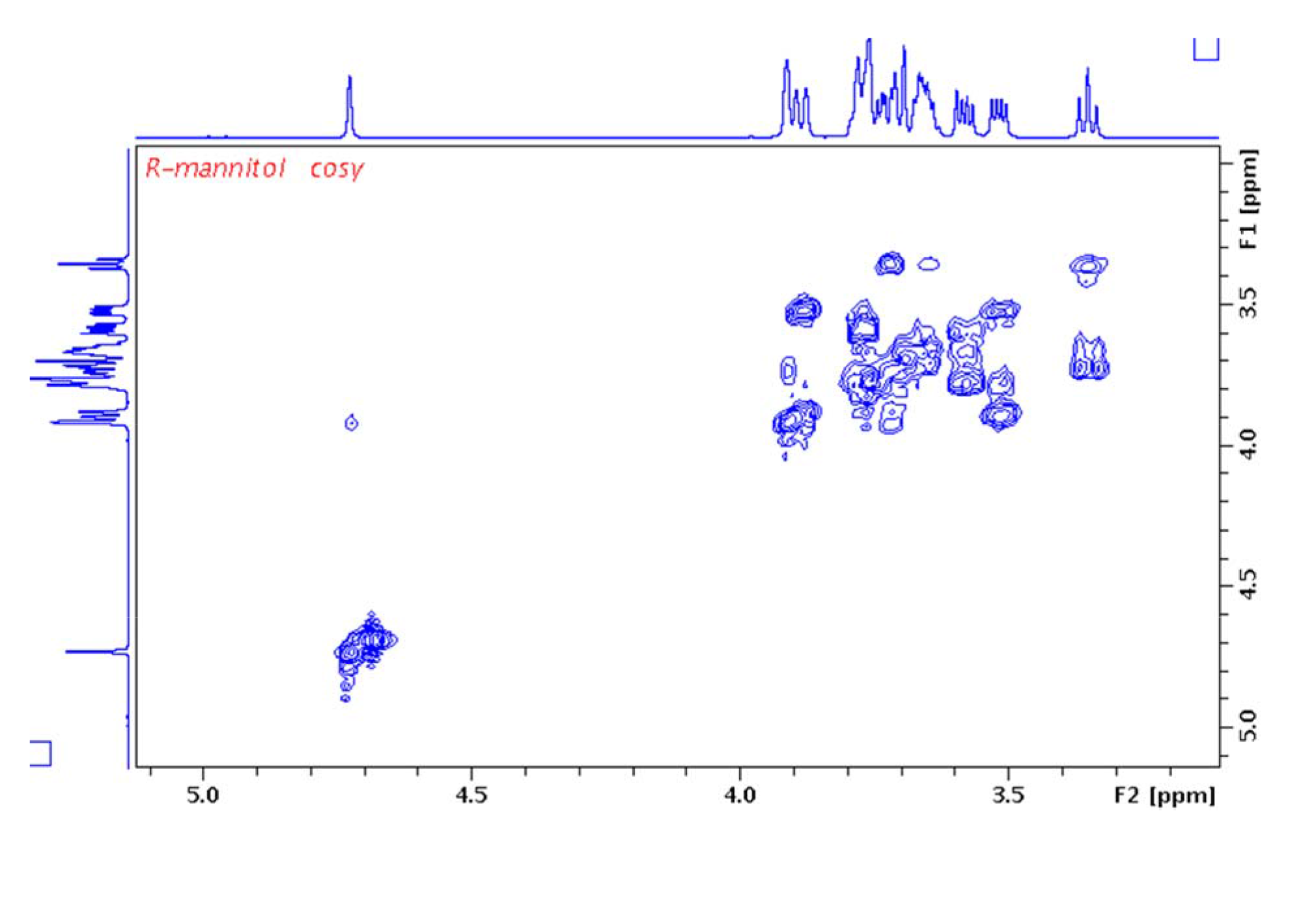

Supplement: S6 Fig — (TIF) [file pone.0140531.s006.tif]

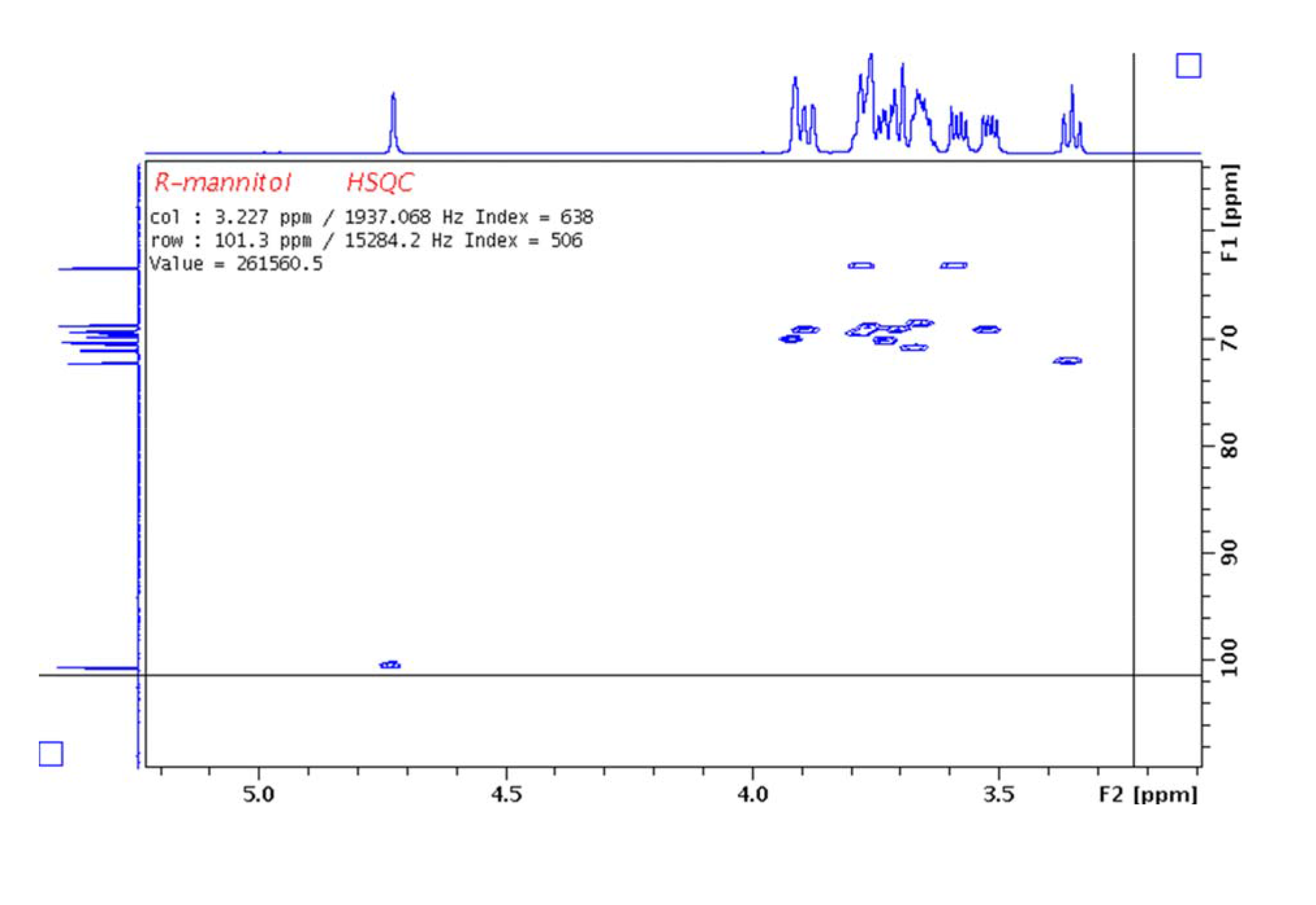

Supplement: S7 Fig — (TIF) [file pone.0140531.s007.tif]

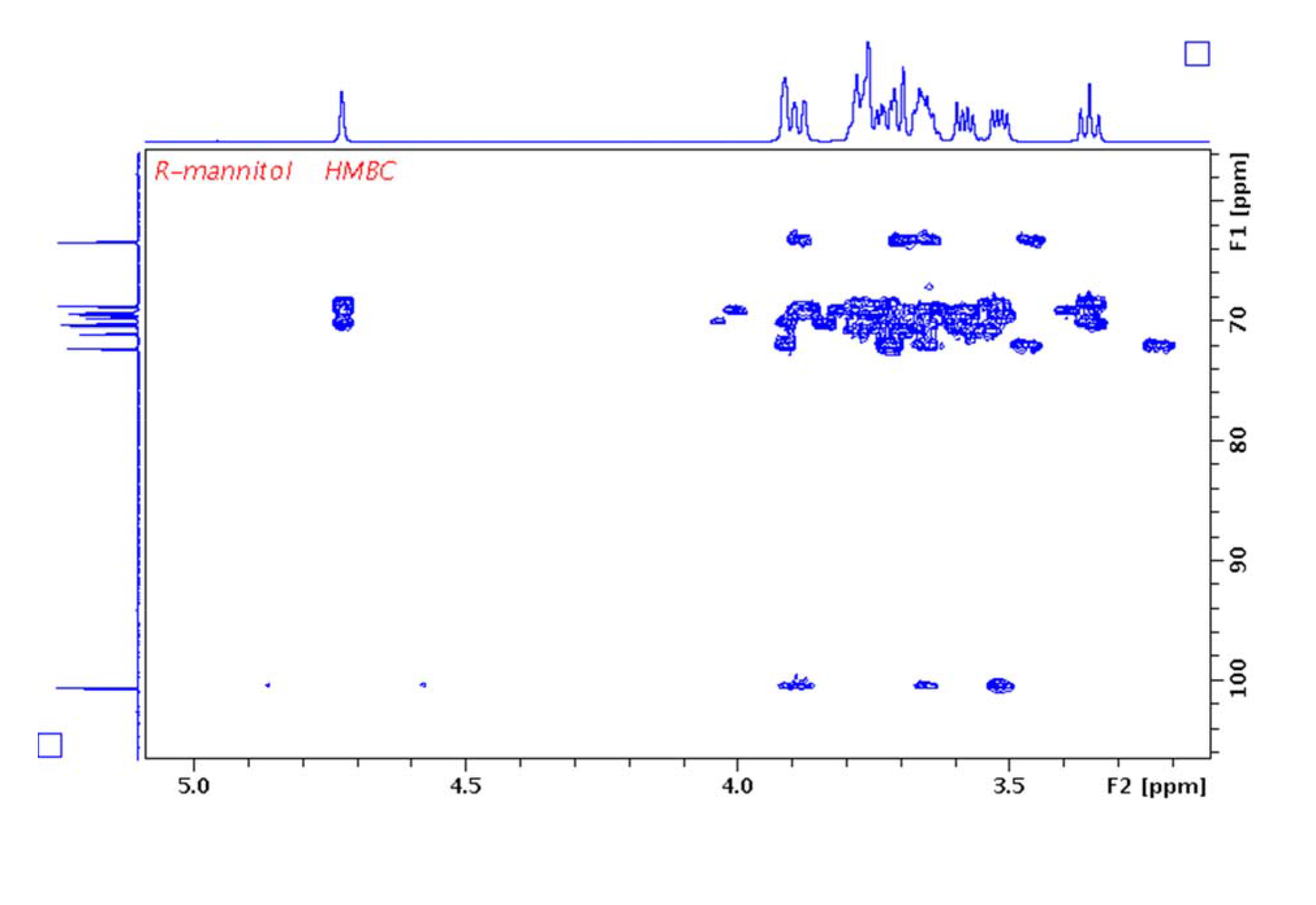

Supplement: S8 Fig — (TIF) [file pone.0140531.s008.tif]

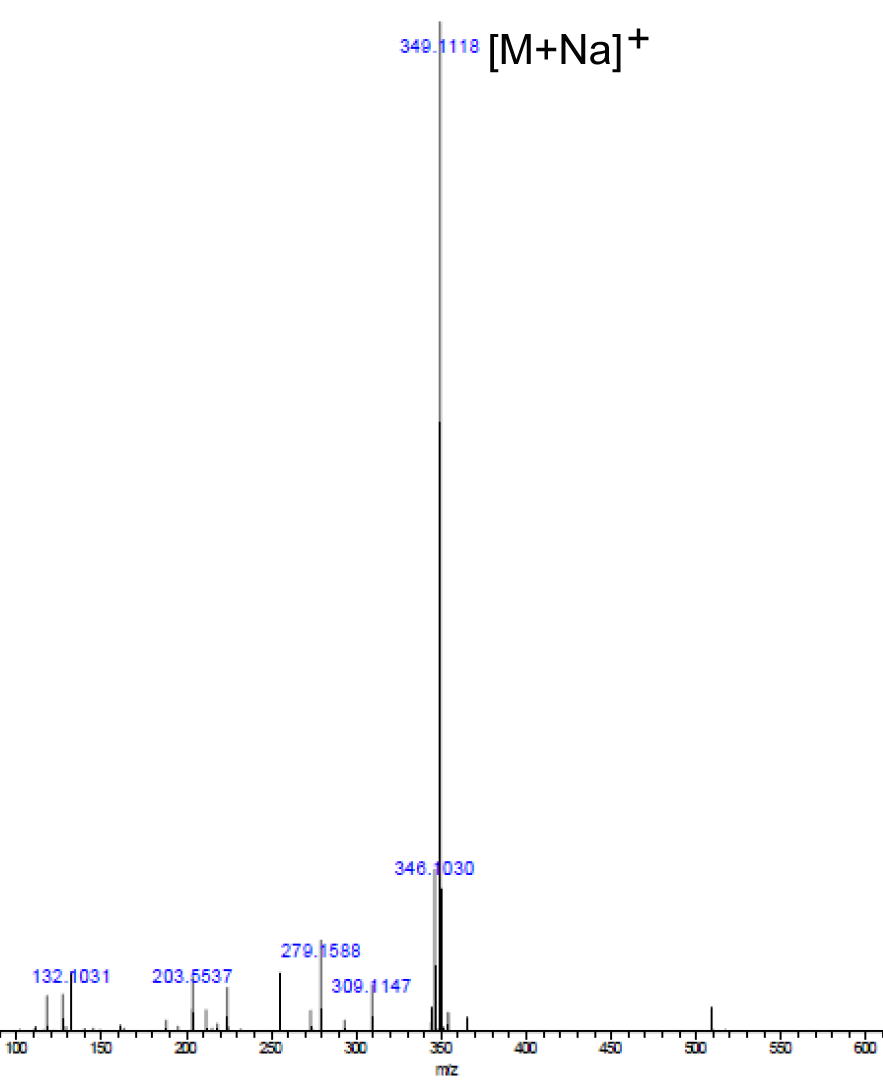

Supplement: S9 Fig — (TIF) [file pone.0140531.s009.tif]

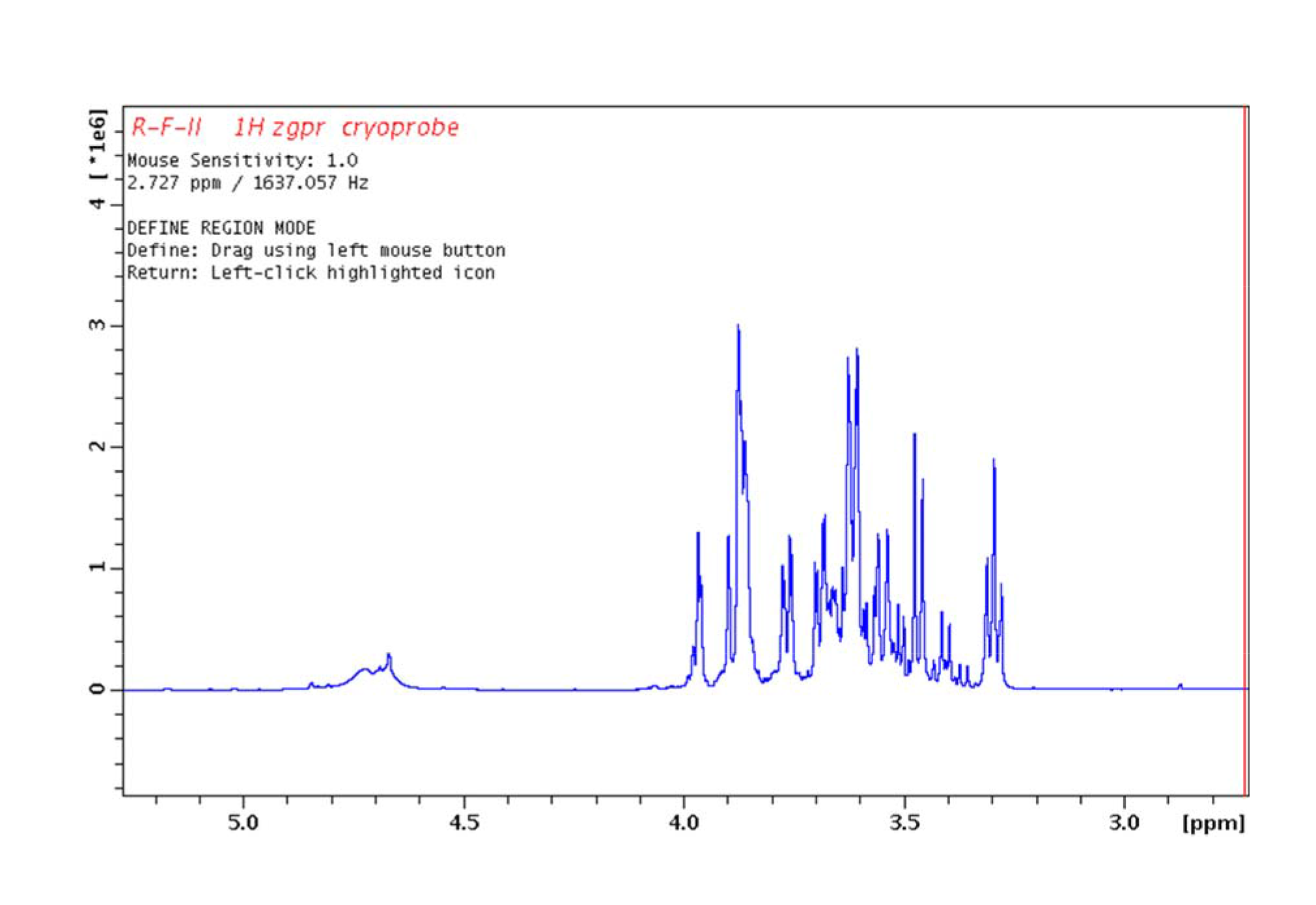

Supplement: S10 Fig — (TIF) [file pone.0140531.s010.tif]

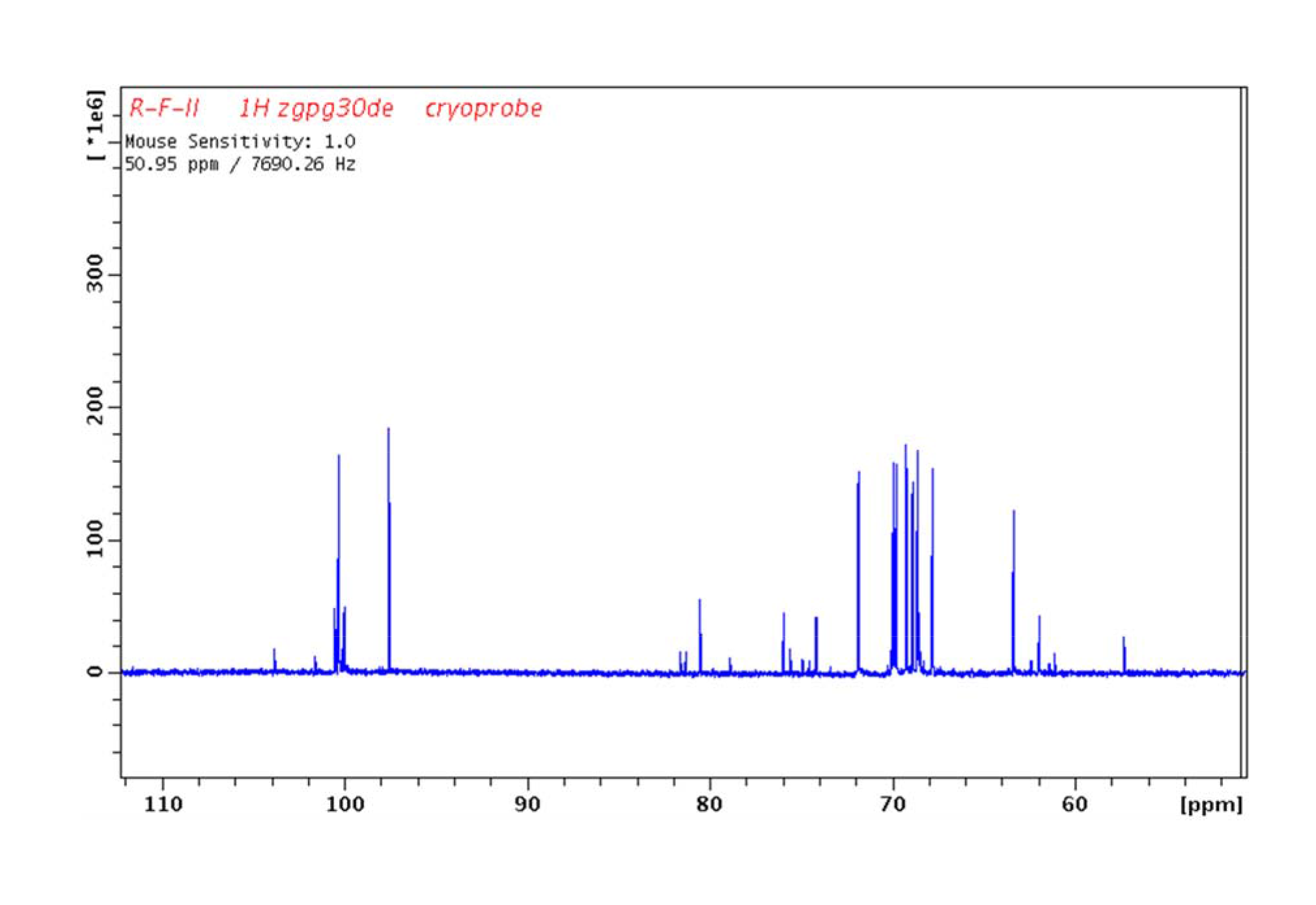

Supplement: S11 Fig — (TIF) [file pone.0140531.s011.tif]

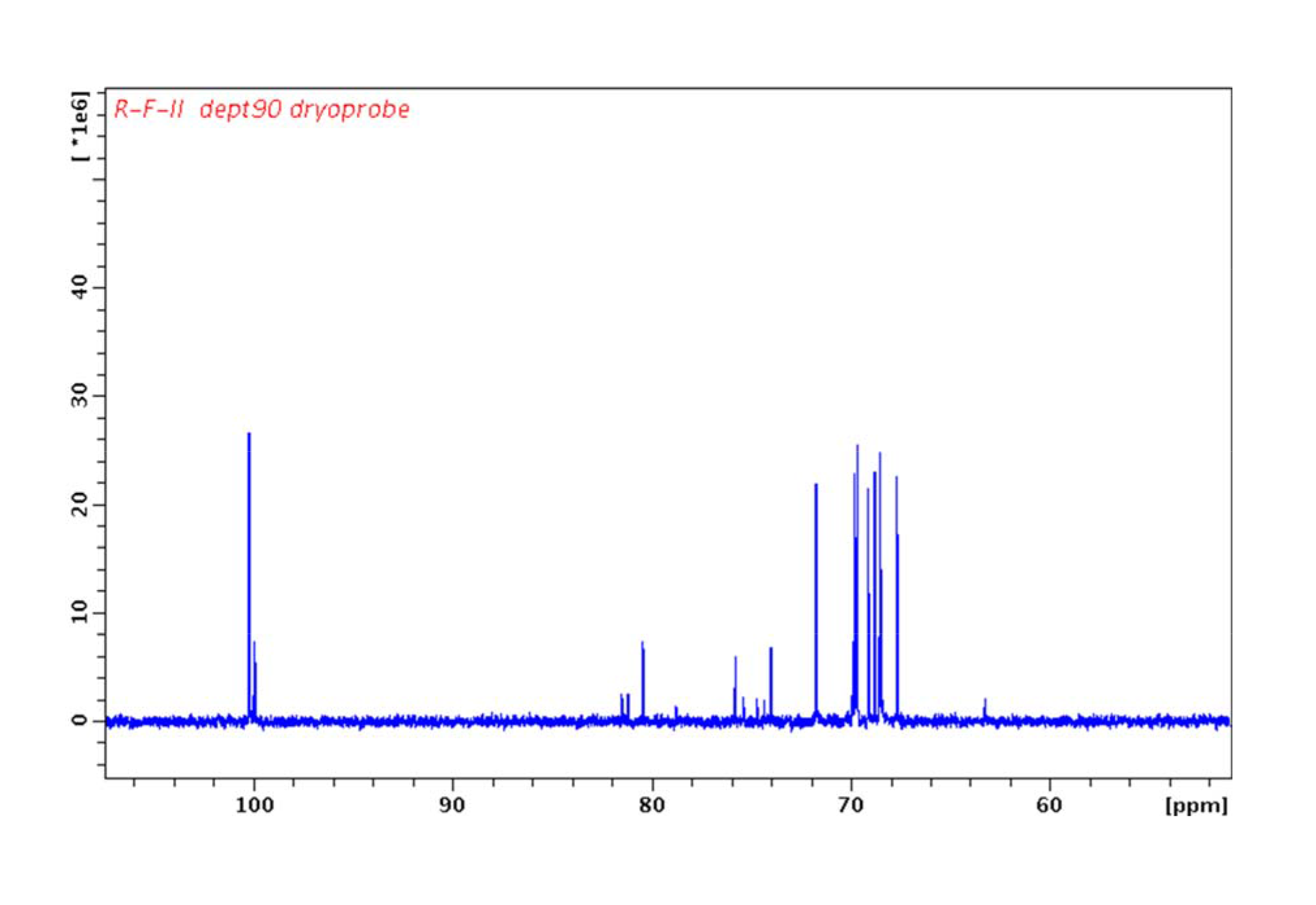

Supplement: S12 Fig — (TIF) [file pone.0140531.s012.tif]

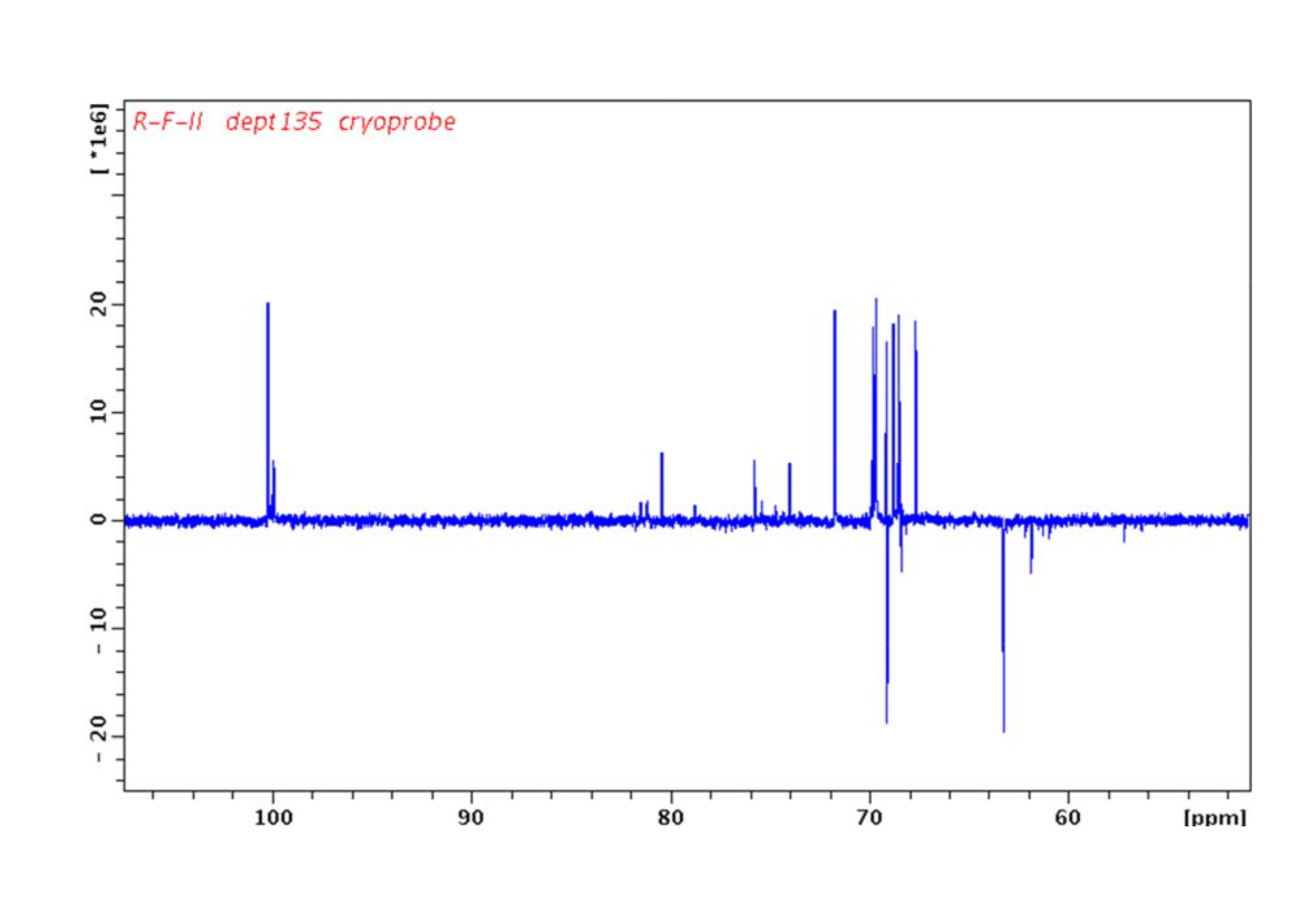

Supplement: S13 Fig — (TIF) [file pone.0140531.s013.tif]

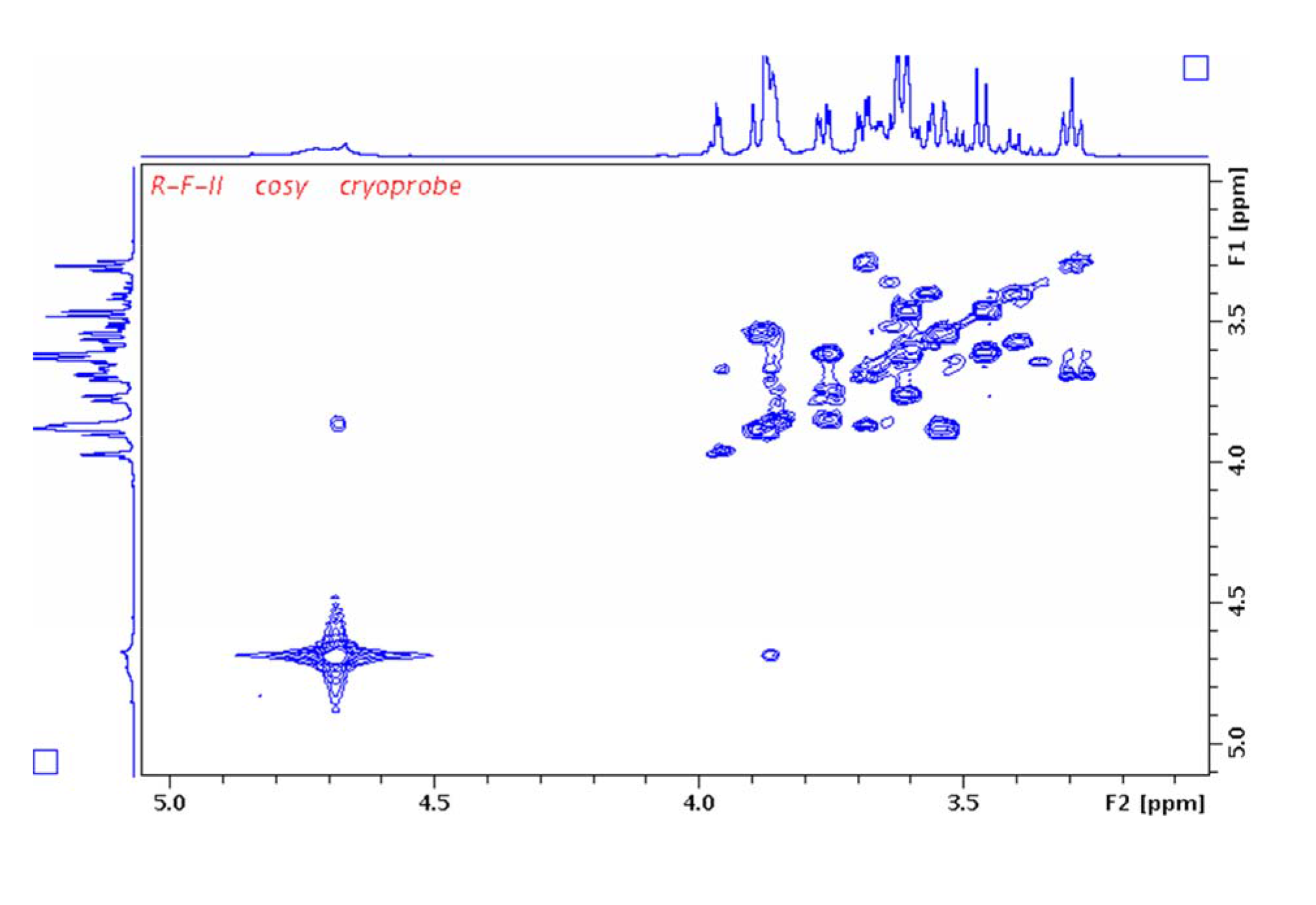

Supplement: S14 Fig — (TIF) [file pone.0140531.s014.tif]

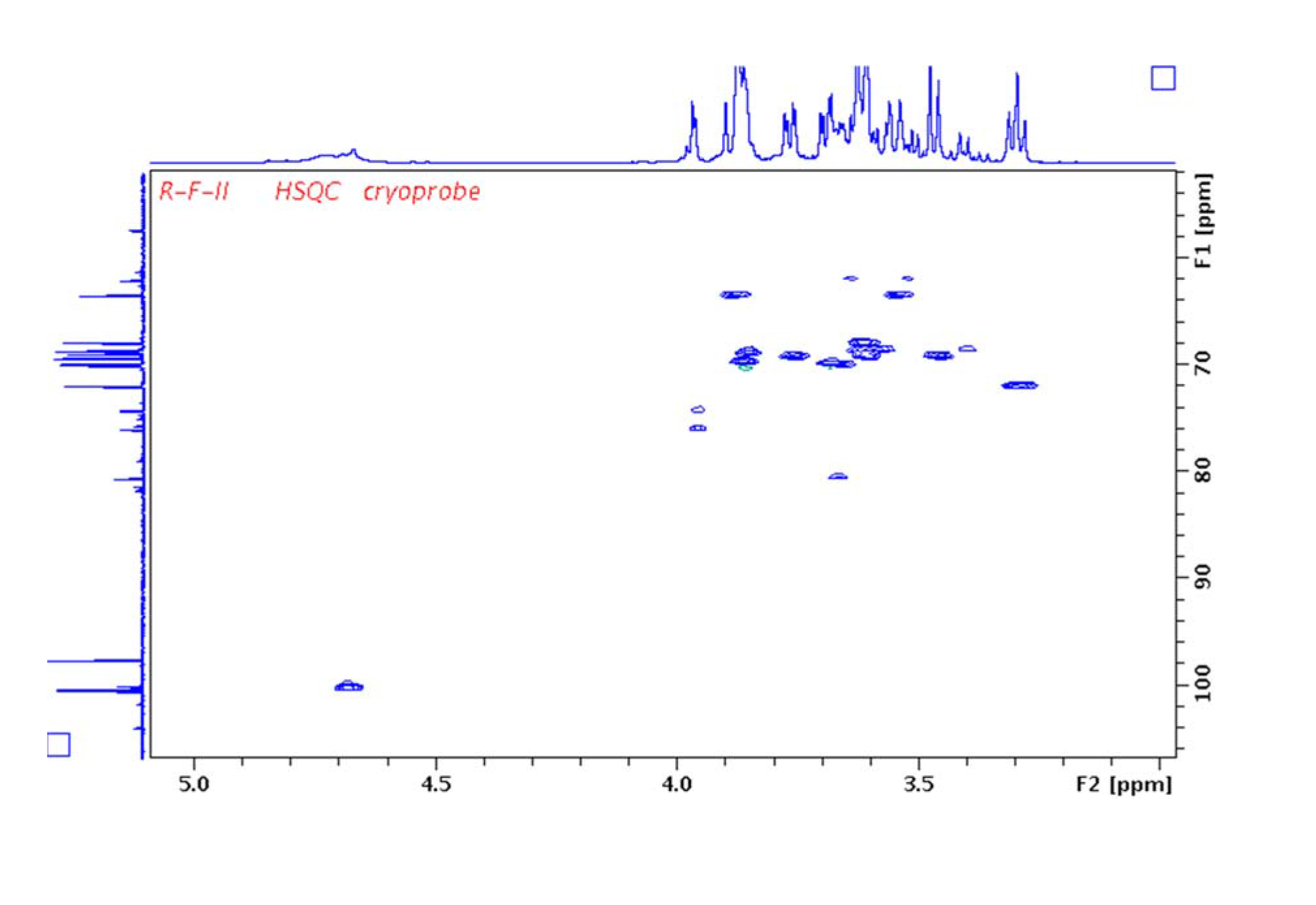

Supplement: S15 Fig — (TIF) [file pone.0140531.s015.tif]

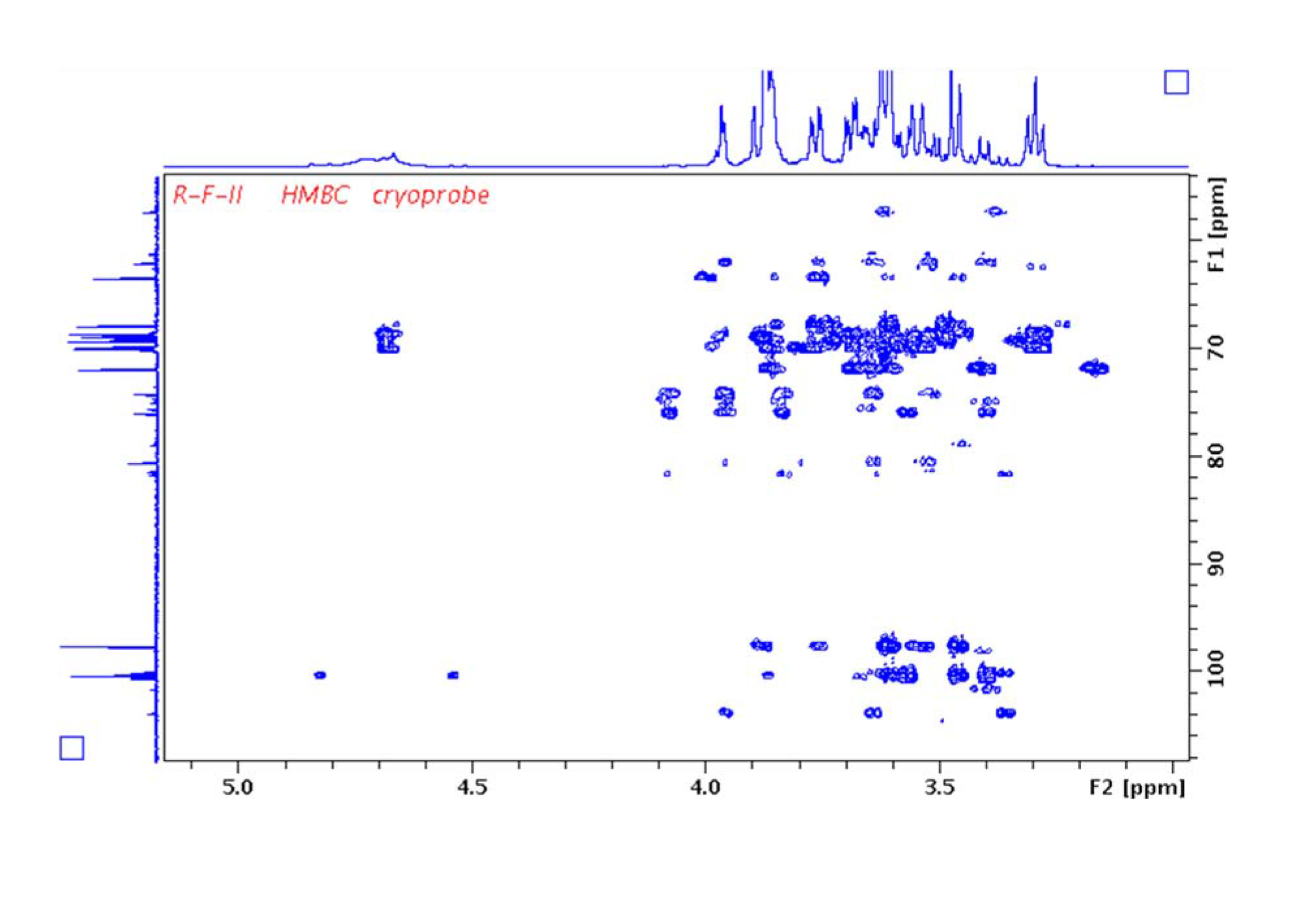

Supplement: S16 Fig — (TIF) [file pone.0140531.s016.tif]

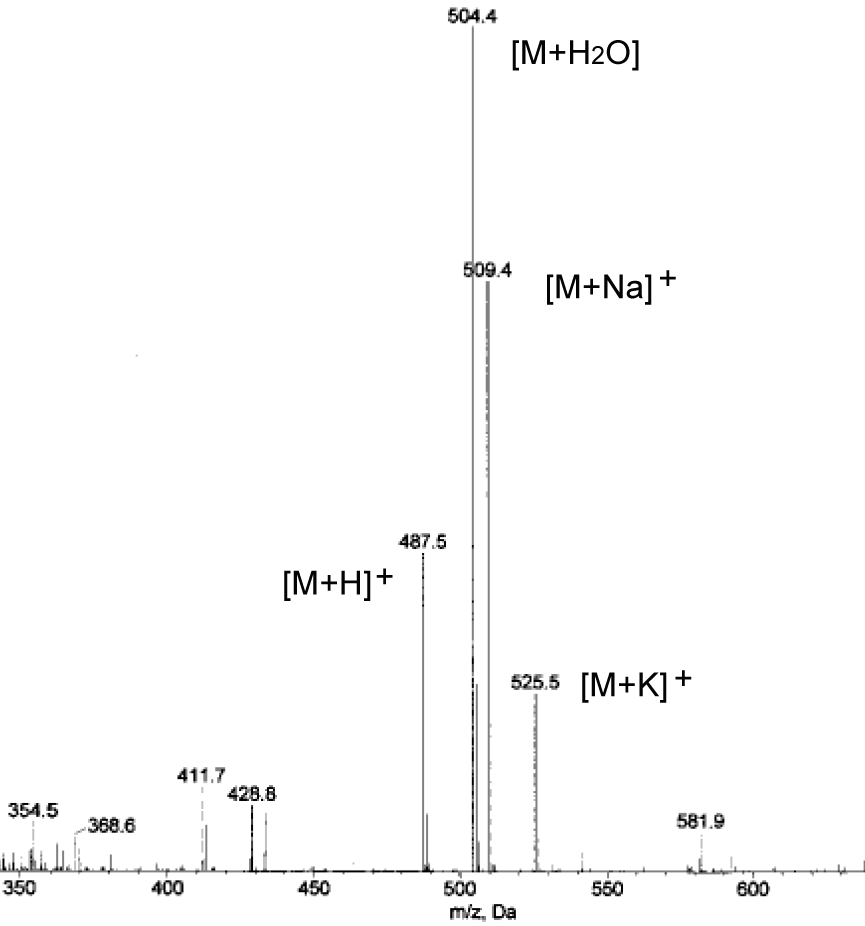

Supplement: S17 Fig — (TIF) [file pone.0140531.s017.tif]

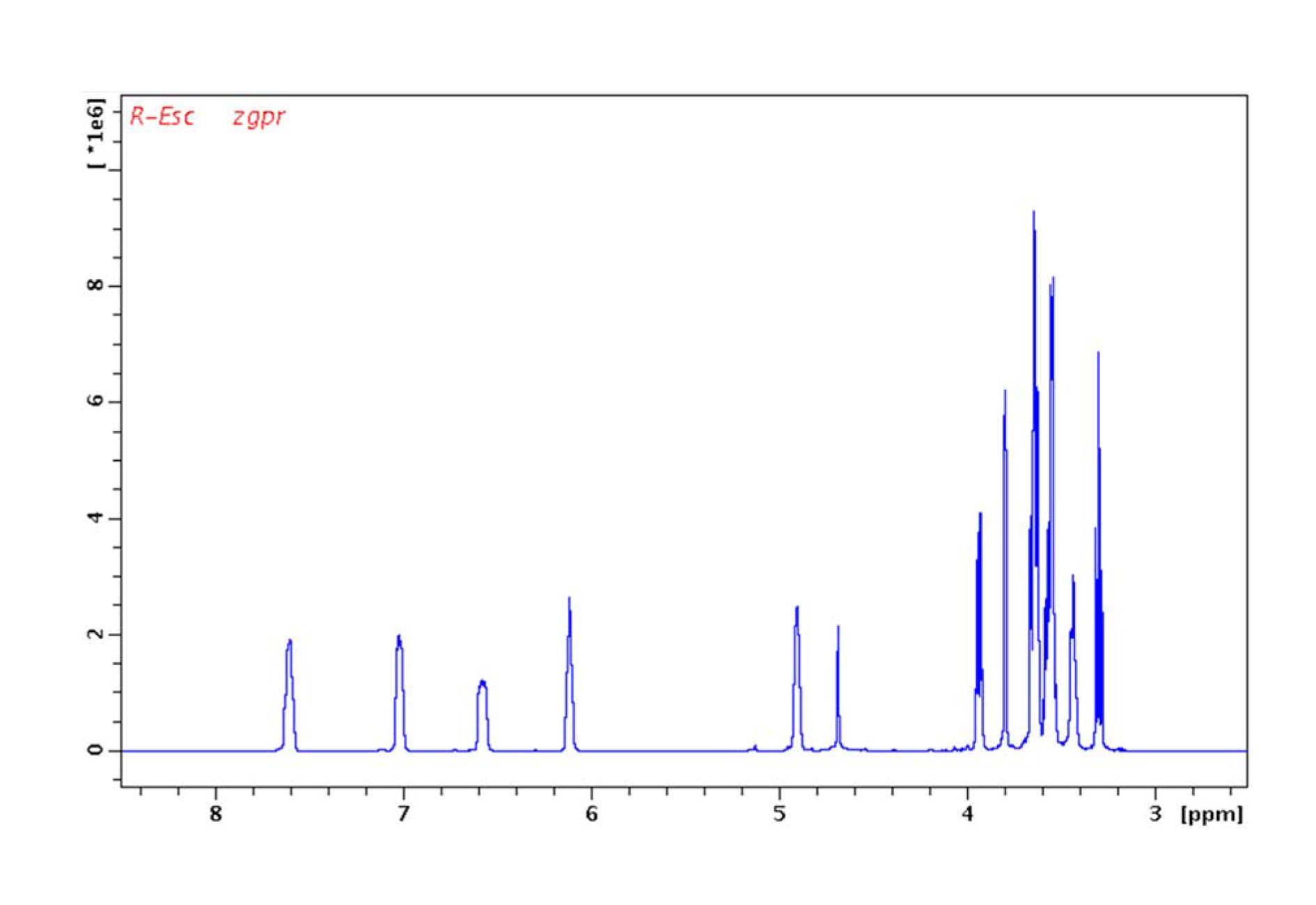

Supplement: S18 Fig — (TIF) [file pone.0140531.s018.tif]

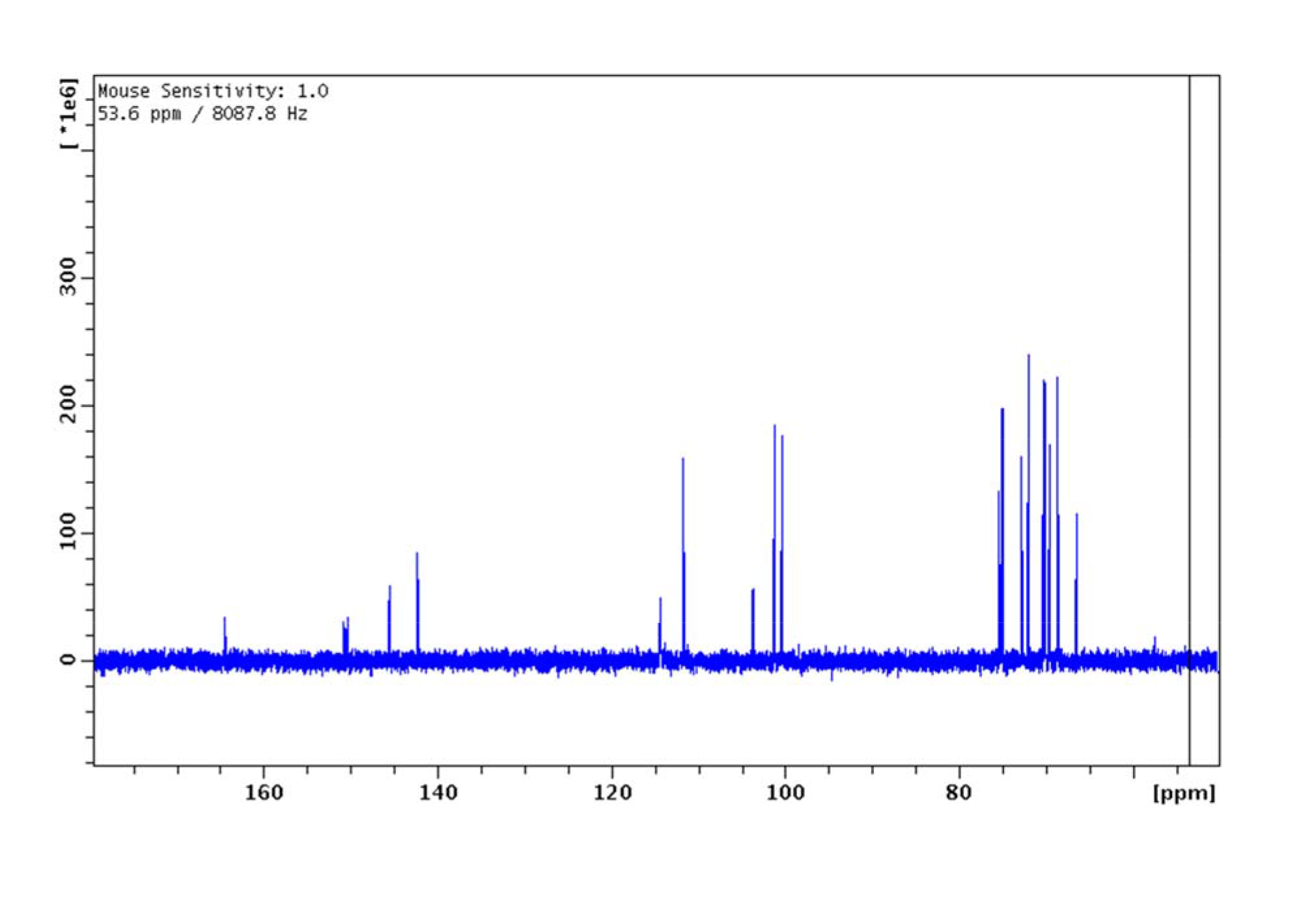

Supplement: S19 Fig — (TIF) [file pone.0140531.s019.tif]

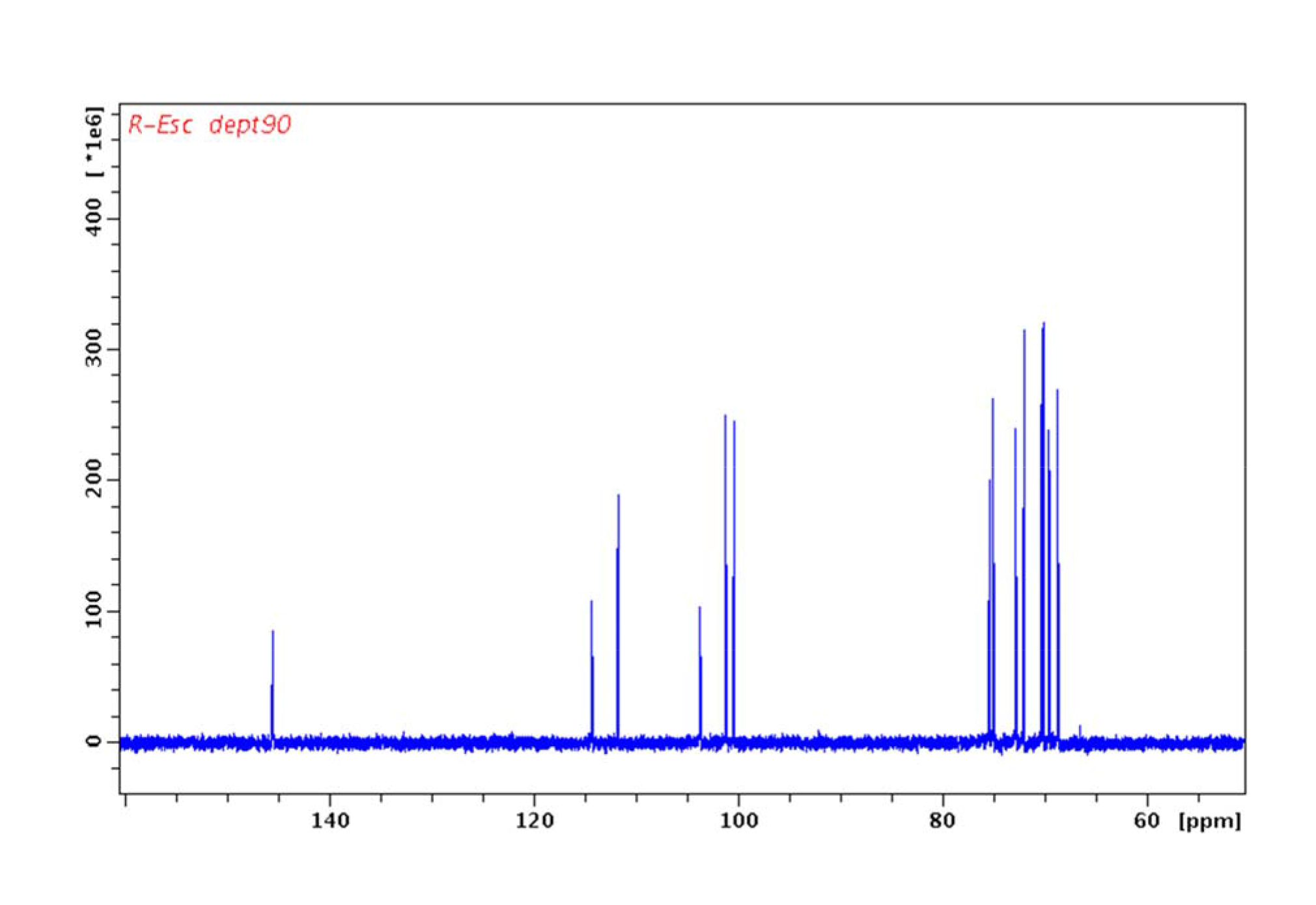

Supplement: S20 Fig — (TIF) [file pone.0140531.s020.tif]

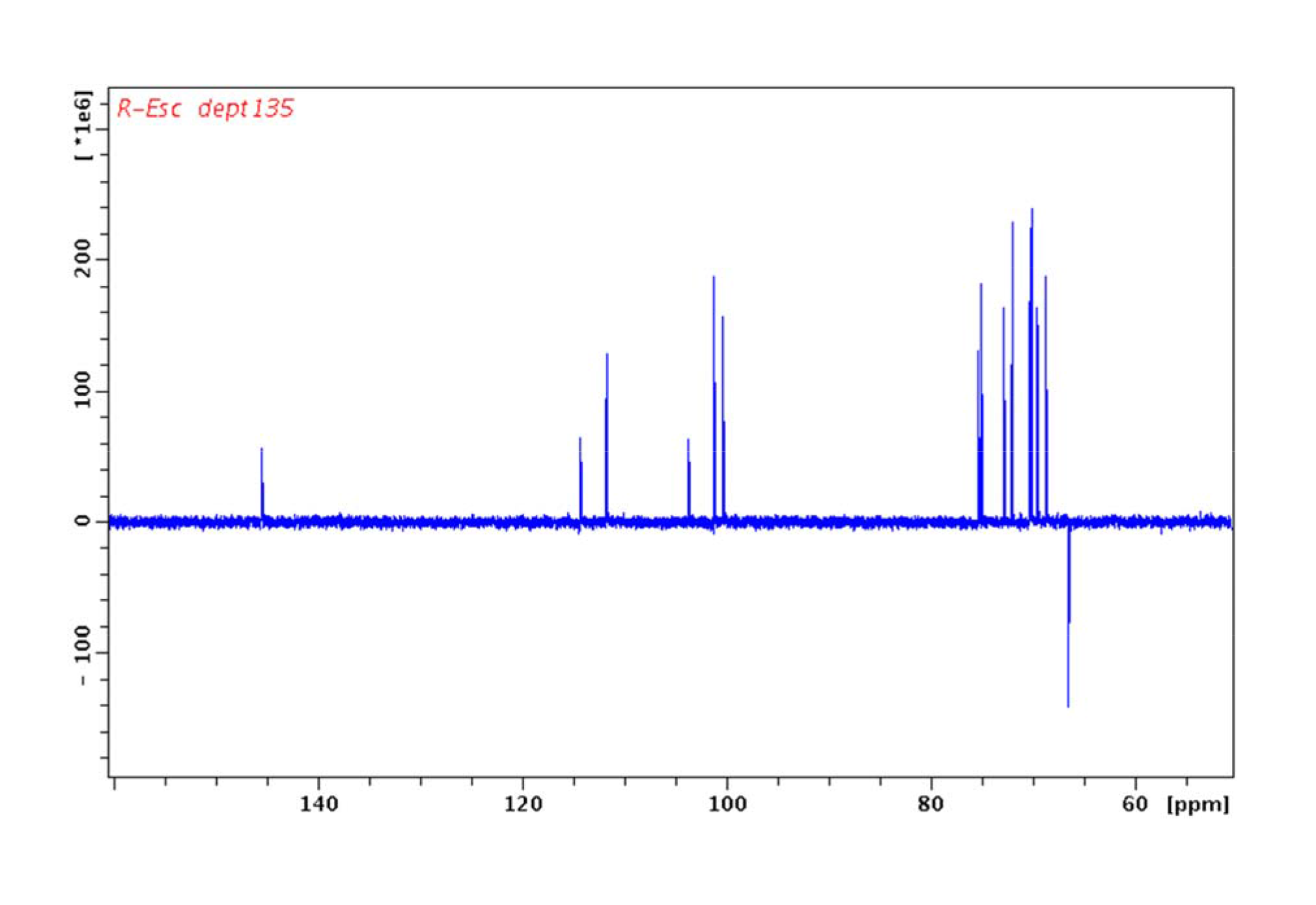

Supplement: S21 Fig — (TIF) [file pone.0140531.s021.tif]

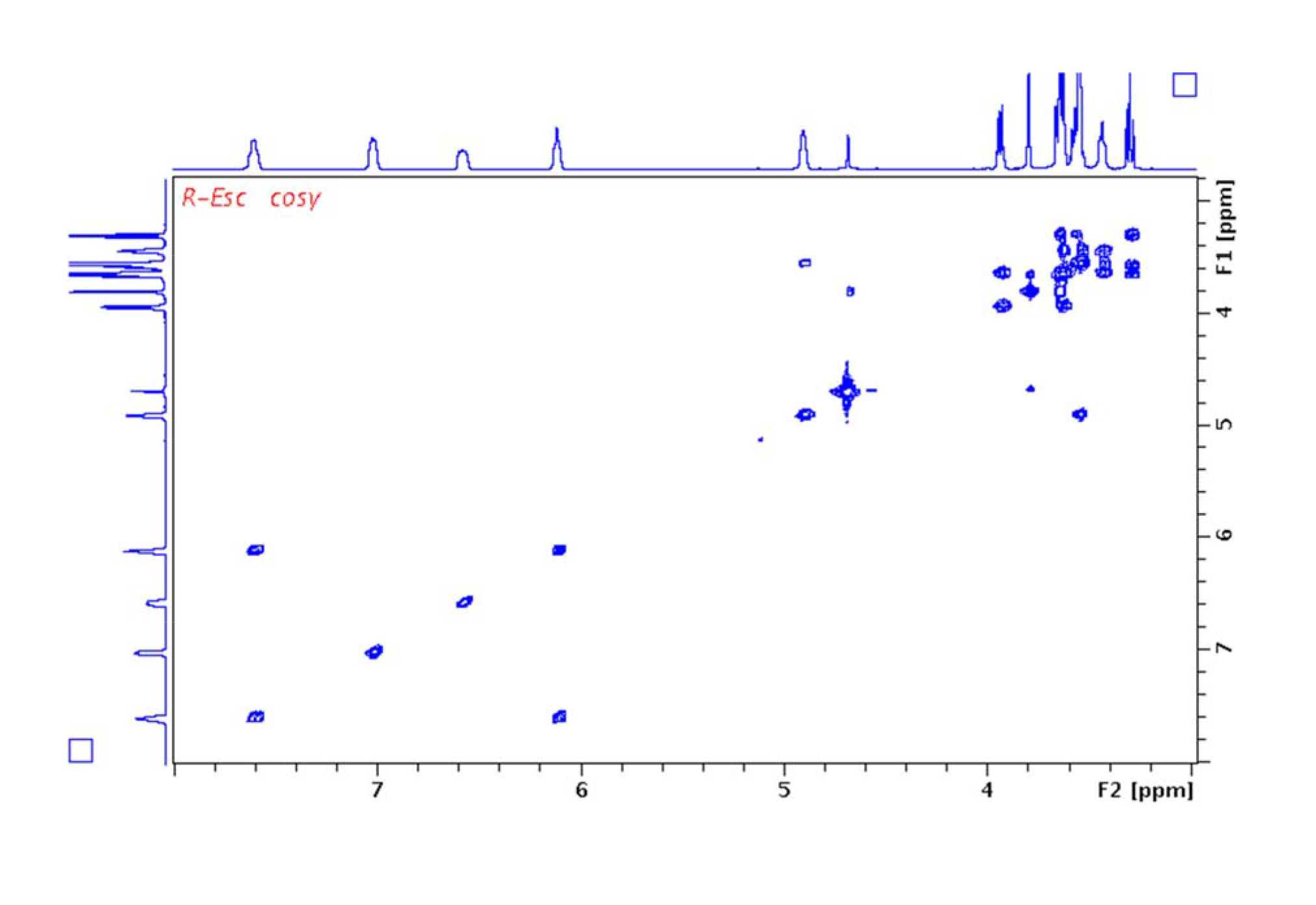

Supplement: S22 Fig — (TIF) [file pone.0140531.s022.tif]

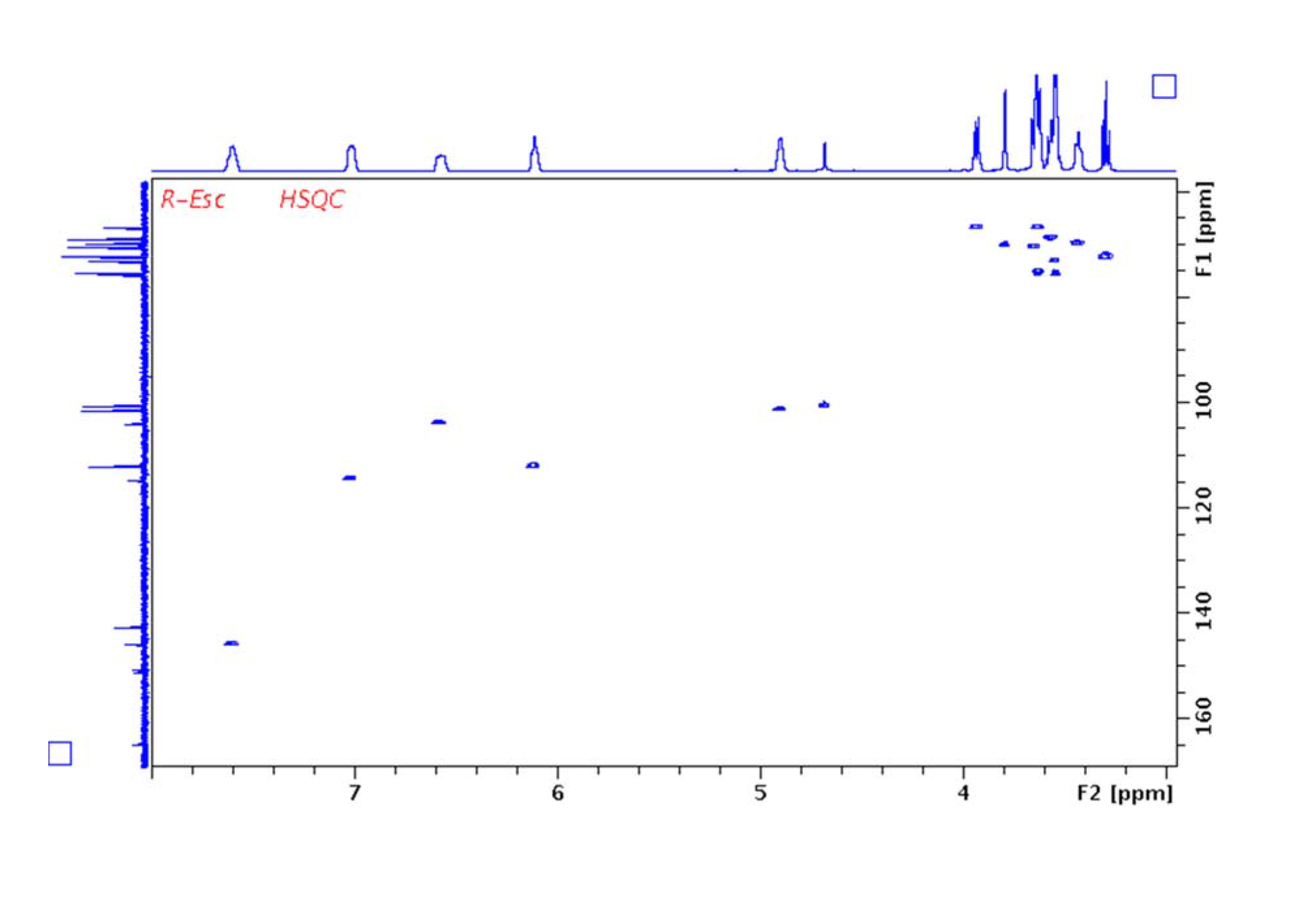

Supplement: S23 Fig — (TIF) [file pone.0140531.s023.tif]

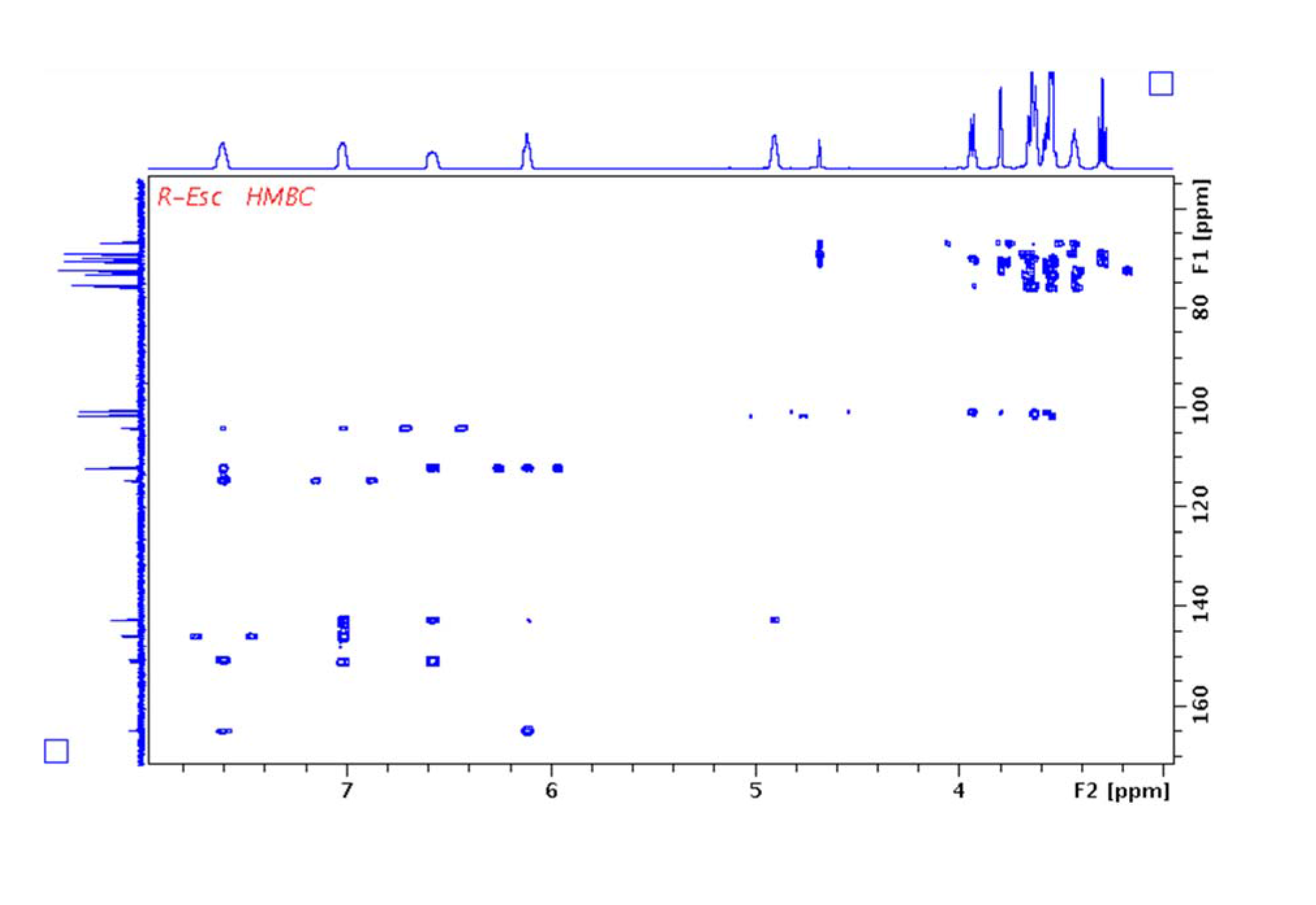

Supplement: S24 Fig — (TIF) [file pone.0140531.s024.tif]
